# Gene fusion and functional diversification of *P450* genes facilitate thermophilic fungal adaptation to temperature change

**DOI:** 10.1080/21501203.2024.2324993

**Published:** 2024-04-02

**Authors:** Shuhong Li, Jiangbo He, Qunfu Wu, Jianghui Gou, Donglou Wang, Xuemei Niu

**Affiliations:** a State Key Laboratory for Conservation and Utilization of Bio-Resources in Yunnan, School of Life Sciences, Key Laboratory for Microbial Resources of the Ministry of Education, Yunnan University, Kunming, China; b Kunming Key Laboratory of Respiratory Disease, Kunming University, Kunming, China

**Keywords:** *P450s*, *Thermomyces dupontii*, gene fusion, prenylated indole alkaloid, temperature changes, Fe^2+^/Fe^3+^ ratios

## Abstract

Thermomyces dupontii harbors two *P450* paralogs (*P450S* and *P450L*) in the gene cluster for the biosynthesis of prenylated indole alkaloids (PIAs) and correponding iron chelators with *P450L* assigned as one protein containing a CYP like domain fused with a FAD-binding domain-containing oxidoreductase. Genetic manipulation and metabolic profile analysis indicated both *P450S* and *P450L* were involved in transforming simple PIAs to their corresponding iron chelators. Moreover, *P450S* is responsible for bolstering simple PIAs to complex PIAs, and *P450L* for reinforcing conjugating unsaturated systems in complex PIAs. Chemical investigation led to isolation and characterization of novel complex PIA metabolites with more oxidations. *P450L* also contributed to forming the third iron-chelating core in iron chelators. A series of iron bioassays and infrastructure analysis revealed that lack of these *P450* genes caused strongly elevated Fe^3+^ levels but attenuated Fe^2+^ levels, together with abnormal mitochondria in mycelia and lipid droplets and vacuoles in conidia. Phenotype analysis revealed that *P450S* and *P450L* facilitated fungal colony pigments, conidial formation and germination via bolstering conidiophores and cell walls in response to temperature reduction.

## 1. Introduction

The diversity in chemical structures is driven by large gene families whose products carry out enzymatic reactions in structural modification and diversification of one specific type of metabolites (Walsh and Moore 2019). Cytochrome *P450* (*P450*) genes are one of the largest and most diverse superfamilies of genes encoding haem-containing monooxygenases found in all kingdoms of life (Manikandan and Nagini 2018; Urlacher and Girhard 2019; Zhang et al. 2021). The functions of *P450* genes are highly variable and organism-dependent. *P450* genes in plants, fungi, and bacteria have been assumed to be widely used for biosynthetic transformations in secondary metabolism, while in mammals and humans for hormone biosynthesis and xenobiotic metabolism (Manikandan and Nagini 2018; Urlacher and Girhard 2019; Zhang et al. 2021). The diversity and diversification of this family of genes are of paramount importance in the great enrichment of the diversity and complexity of secondary metabolites. However, the biological and ecological importance of these *P450*-mediated metabolites in fungi remains largely cryptic.

Cytochrome *P450* genes are frequently located close to a cyclodipeptide synthase gene in microbes (Li 2010; Xu et al. 2014; Giessen and Marahiel 2015; Canu et al. 2020). Cyclodipeptides are an important family of iron-chelating precursors in bacteria. The nearby *P450* gene is primarily involved in oxidising the diketopiperazine nitrogen atoms in cyclodipeptide to be the corresponding N-oxides, resulting in two hydroxamate cores for chelating iron (Cryle et al. 2010; Giessen and Marahiel 2015; Canu et al. 2020). For instance, the cytochrome *P450* gene *CYP134A1* from *Bacillus subtilis* is responsible for transforming cyclodipeptide cyclo-leu-leu into pulcherriminic acid, a precursor of an extracellular iron chelate pulcherrimin (Cryle et al. 2010). These cyclodipeptide-derived iron chelators are involved in sequestering Fe^3+^ and play a crucial role in bacterial growth and survival. However, the ecological function of *P450*-mediated iron chelators in bacterial adaption to temperature changes remains largely unknown.

In fungi, cyclodipeptides were commonly synthesised from tryptophan and a small group of amino acids (glycine, alanine, proline, and its derivatives). These cyclodipeptides were further prenylated to form prenylated indole alkaloids (PIAs) (Li 2010; Giessen and Marahiel 2015). PIAs are a well-known class of fungal metabolites with a wide range of biological and pharmacological activities and are widely distributed in filamentous fungi, especially in the genera *Penicillium* and *Aspergillus* of Ascomycota (Li 2010; Giessen and Marahiel 2015). One *P450* gene is supposed to be involved in forming two hydroxamate cores in the PIAs, such as astechrome from *Aspergillus terreus* and hexadehydroastechrome from human pathogen *Aspergillus fumigatus*, which are two well-known trimeric complexes of PIA derivatives with Fe^3+^ (Arai et al. 1981; Yin et al. 2013; Haas 2014; Bok et al. 2015). Another *P450* gene is believed to be responsible for forming one pyrano ring to yield complex prenylated pyranoindole alkaloids (PPIAs), which is the remarkable difference in PIAs between common fungi and extremophilic fungi (Kato et al. 2007; Chu et al. 2010; Ding et al. 2010; Finefield et al. 2011; Guo et al. 2011). From a structural point of view, the extra pyran ring fused with the indole core leads to the formation of a large conjugated unsaturated system, which contributes to more stable structures of the compounds. However, the specific *P450* genes and their chemical functions in the PPIA biosynthetic pathways remained unclear.

Thermophilic fungi are eukaryotes that can grow at the high temperatures of 45–60 °C and are often found as the chief components of the mycoflora in a variety of natural and manmade composting systems, including rotting hay, stored grains, wood mulch, nesting material of birds and animals, municipal refuse, and self-heating accumulated organic matter (Maheshwari et al. 2000; Chen et al. 2023; Yang et al. 2023). *Thermomyces* is a predominant thermophilic fungal genus that is phylogenetically closely related to the common mesophilic fungi *Aspergillus* and *Penicillium* genera (Houbraken et al. 2014). *Thermomyces* species are ideal models for elucidating the underlying evolution mechanism of fungal adaption to temperature changes due to their sensitivity to cold stress and small genome sizes (Chen et al. 2023; Yang et al. 2023). Our previous study demonstrated that *Thermomyces dupontii* could produce a type of potent nematocidal polyketide synthetase-nonribosomal peptide synthetase (*PKS-NRPS*) thermolides A–B with an unprecedented chemical skeleton containing a 13-membered lactam bearing macrolactone, quite similar to those from a bacterial origin (Guo et al. 2012). These studies suggest that *T. dupontii* harbours unprecedented biosynthetic mechanisms for these unique secondary metabolites via dramatically reduced genes and clusters.

**Figure 1.**
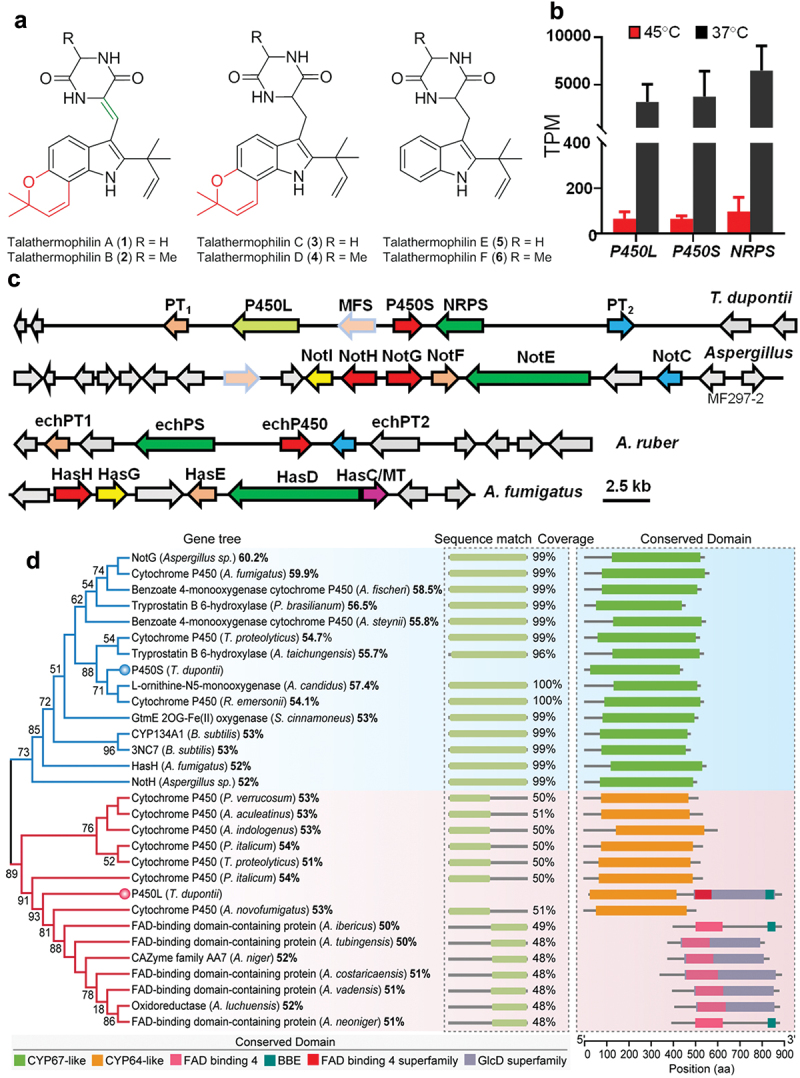
(a) The structures of talathermophilins (1–6), including four complex pyran prenylated indole alkaloids (PPIAs) with pyrano ring (in red) (1–4) and two simple prenylated indole alkaloids (PIAs) (5–6), were accumulated in *Thermomyces dupontii* at minimum growth temperature 37 °C. (b) Comparison of the transcriptional levels of two *P450* genes, *P450S* and *P450L*, together with *NRPS* gene, in *T. dupontii* at 37 °C *vs*. 45 °C. (c) Schematic view of the organization of the *pia* gene cluster in *T. dupontii* that consisted of 5 key biosynthetic genes with their predicted functions color-coded and their homologues in the other fungal species that had similar key genes (similar functions in the same colors). No homologues for the large *P450L* in *T. dupontii* were found in the *pia* gene clusters in *Aspergillus* sp. (d) Phylogenetic analysis of *P450S* and *P450L* in the *pia* gene cluster from *T*. *dupontii*, and their homologs, and other genes with relative functions in other fungi based on their amino acid sequences and the predicted domains for each full-length amino acid sequence. Numbers at nodes represent the bootstrap values of the neighbor-joining tree. *P450L* from *T*. *dupontii* contained two quite different domains. MT, methyltransferase. All the genes only encoded one enzyme with one domain, such as NotG-NotI from *Aspergillus* sp. were assigned as CYP67-like, CYP503A1-like, and UbiH/FAD-dependent oxidoreductases, respectively; HasG-HasH from *Aspergillus fumigatus* were assigned as GlcD/FAD-binding oxidoreductase, and CYP_GliC-like respectively; and EchP450 from *A. ruber* was assigned as CYP64-like.

Our recent studies have shown that *T. dupontii* strains at 37 °C accumulate a class of PIAs, talathermophilins A–F (1–6) (Figure 1a), including four complex PPIAs with pyrano ring (1–4) and two simple PIAs (1–2) (Chu et al. 2010; Guo et al. 2011; Chen et al. 2023). Three types of PIAs have been found in *T. dupontii* according to the patterns of prenylation and oxidation. Among them, complex PPIAs 1–4 share the same key pyranoindole and the same oxidation pattern as a key versatile precursor notoamide E that has long been proposed in the biosynthetic pathways for the well-known complex PPIAs in a marine *Aspergillus* species (Kato et al. 2007; Chu et al. 2010; Ding et al. 2010; Finefield et al. 2011; Guo et al. 2011). These findings suggest that the thermophilic fungus *T. dupontii* can produce much more stable key metabolites that could complement metabolite libraries of fungi that live at common temperatures.

Bioinformatics analysis suggested that *T. dupontii* had only one *NPPS*-terpenoid hybrid gene cluster (*PIA* cluster), which contained one *NRPS* gene, two prenyltransferase genes, and two *P450* genes, and was putatively involved in the formation of talathermophilins (1–6). Our most recent study revealed that disruption of the *NRPS* gene could result in the absence of the whole talathermophilin biosynthetic pathway starting with tryptophan and either glycine or alanine in the mutant *ΔNRPS* (Chen et al. 2023). However, how these two *P450* genes were involved in the formation of the key pyranoindole and oxidation patterns of talathermophilins in thermophilic fungi remains unknown. How these two *P450* genes were involved in talathermophilin-derived iron chelators remains unknown. Moreover, *ΔNRPS* lacked the rarely thick and strong conidiophores that appeared in WT at relatively low temperatures (Chen et al. 2023). Whether these two *P450* genes contributed to the formation of the unprecedented conidiophores also remained unclear.

To gain insight into the underlying mechanisms of the P450-mediated chemical functions in the fungal adaption to temperature changes, here we have applied bioinformatics, genetic manipulation, metabolic profiles, and chemical investigation to uncover fundamental differences in the *P450* gene-mediated metabolomic adaptation of *T. dupontii* to temperature change from fungal optimal growth at 45 °C to its minimum growth at 37 °C. Further morphological phenotype and infrastructural analysis of two mutants and WT, combined with iron bioassays, were conducted to characterise the biological functions of P450-mediated iron chelators in fungal adaption to temperature changes.

## 2. Experimental procedures

### 2.1. Organisms, plasmids, and culture conditions

*Thermomyces dupontii* YM3–4 was obtained from the State Key Laboratory for Conservation and Utilization of Bio-Resources & Key Laboratory for Microbial Resources of the Ministry of Education. *Escherichia coli* strain DH5α (Tsingke, Kunming, China) was used to construct and store recombinational plasmids. All fungal strains were cultivated on potato dextrose agar (PDA): potato (Kunming, China) 200 g/L, glucose (Solarbio, Beijing, China) 20 g/L, and agar 15 g/L. About 9 mm inoculas of fungal strains were cultured on 9 cm diameter glass Petri dishes containing PDA plates at 45 °C for 7 days. Fungal media PDA was used for analysis of the colony growths and phenotypic traits of the fungal strains cultivated at 45 °C and 37 °C.

### 2.2. Bioinformatics analysis

The compute pI/*M*_w_ tool of the ExPASy database was applied for the evaluation of two P450 proteins. The functional domains of the two proteins were analysed using the InterProScan database; We compared and downloaded the top 100 annotated protein sequences in the NCBI database with annotation functions and sequence alignment consistency greater than 50%. The neighbour-joining estimate in MEGA6 software was used to construct the protein sequence phylogenetic evolutionary tree of the *P450L* (*GME4767_g*) and *P450S* (*GME4769_g*). RNA-seq data was mapped to the *T. dupontii* YM3-4 genome using STAR (v 2.7.10b) with default parameters (Dobin et al. 2013). The output was sent to “samtools sort” to produce a sorted bam file (Danecek et al. 2021). Genome-guided Trinity assembly (v2.1.1) (Grabherr et al. 2011) and PASA assembly (v2.3.3) (Haas et al. 2003) were performed to retrieve all transcripts, respectively. Transdecoder (v5.5.0, https://github.com/TransDecoder/TransDecoder/) was used to detect potential ORFs in assembled transcripts. Visualization was performed with the integrative genome browser (IGV) (v2.16.0, http://www.broadinstitute.org/igv/).

### 2.3. Mutant construction via thermophilic CRISPR/Cas9 system

One *SpCas9* expression plasmid *p-TrpC-NLS-coSpCas9-NLS* (Figure S5), which was composed of a selective marker hygromycin (HygB), and the codon-optimised *SpCas9* gene (*coSpCas9*) with nuclear localisation signal sequences (*NLS*) under control of fungal constitutive promoter *TrpC*, were constructed (Nies and Li 2021; Chen et al. 2023; Yang et al. 2023). One sgRNA expression plasmid containing a *tRNA*^*Gly*^ self-processing system with a native promoter *ptRNA*^*Gly*^ from *T*. *lanuginosus* was also used. The protospacer and the corresponding PAM sequences for each sgRNA were chosen from the 5’ part of the putative catalytic domain in each target gene and were listed in Table S3. Each sgRNA sequence was used as a half part of primer that contained overlapping 20 bp from the sgRNA expressing plasmid *p-tRNA*^*Gly*^*-sgRNA* carrying one *tRNA*^*Gly*^ system containing a promoter *ptRNA*^*Gly*^ used in *T*. *dupontii* (Figure S5). The sgRNA expression plasmids for the target gene editing in *T*. *dupontii* were thus constructed through PCR and In-Fusion methods (Labuhn et al. 2018). Then the SpCas9 expressing plasmid *p-TrpC-NLS-coSpCas9-NLS* and the sgRNA expression plasmid were transformed into *T*. *dupontii* protoplasts, the selective marker hygromycin B concentration in *T. dupontii* was 200 μg/mL, potential transformed colonies were selected after incubation at 37 °C for 4–6 days.

Medium TB3 [200 g/L sucrose, 3 g/L yeast extract, 3 g/L tryptone, 7.5 g/L agar, and 200 μg/mL hygromycin B (Roche Applied Science, Mannheim, Germany)] was applied to carry out protoplast regeneration for selecting transformants. The 1 mL conidial suspension (1.0 × 10^8^ conidia/mL) from 7 days fungal strains on PDA was inoculated into 150 mL of YPS medium (4 g/L yeast extract, 1.5 g/L soluble starch, 1 g/L KH_2_PO_4_, and 0.5 g/L MgSO_4_·7 H_2_O) and cultured at 45 °C at 180 r/min for 20 h. The mycelia were harvested and then re-suspended in 20 mL [3 mL N-buffer (197.175 g/L MgSO_4_·7 H_2_O, 58.82 g/L Na_3_C_6_H_5_O_7_·2 H_2_O, pH 5.5 and 17 mL P-buffer (182.1 g/L Sorbitol, 14.7 g/L Na_3_C_6_H_5_O_7_·2 H_2_O, pH 5.8)] filter-sterilised enzyme solution that contained 0.3 g of lysing enzymes (Sigma, St. Louis, MO, USA). The suspension was incubated for 5.5 h at 28 °C on a rotary shaker at 100 r/min. Protoplasts were collected by filtering through six layers of sterile lens-cleaning tissue and centrifuged at 5,000 x *g*. The protoplasts were washed twice with STC [182.1 g/L Sorbitol, 50 mmol/L Tris-HCl (pH 8.0), 7.35 g/L CaCl_2_] solution and finally re-suspended in the same solution.

About 200 μL protoplasts (circa 1.0 × 10^8^/mL) were mixed with 10 μg linear DNA in a 1.5 mL centrifuge tube. After 40 min of incubation on ice, 1 mL of PTC [44.73 g/L KCl, 50 mmol/L CaCl_2_, 50 mmol/L Tris-HCl (pH 8.0), 60% polyethylene glycol 4000] was added into the mixture and mixed gently. After incubation at 45 °C for 30 min and 10 min of incubation on ice, the putatively transformed protoplasts were plated onto TB3 medium (200 μL per plate) containing 200 μg/mL hygromycin B. Transformation colonies were selected after incubation at 45 °C for 2–4 days, and every single colony was transferred to a new plate containing PDA. After incubation for 5 days at 45 °C, the genomic DNA of putative transformants was extracted and verified by PCR to check for the integration of genes in the genome. All the mutants deficient in the target gene were screened out and confirmed by PCR.

### 2.4. Fungal morphology, conidial production, and germination

Mycelium plugs with an estimated 9 mm diameter from 7 days fungal colonies of the wild-type (WT) and mutant strains were inoculated on PDA, TYGA (10 g/L tryptone, 5 g/L yeast extract, 10 g/L glucose, 5 g/L molasses, and 20 g/L agar) and YMA (2 g/L yeast extract, 10 g/L maltose, and 20 g/L agar) medium (5 replicates/strain), and the growth rate and colony morphology were observed during culturing at 45 °C and 37 °C. The diameter of colonies at 45 °C for 3, 5, and 7 days and 37 °C for 6, 9, and 12 days were evaluated. Morphology was observed by staining fresh hyphae under a ZEISS Axioskop 2 Plus fluorescence microscope with calcium fluorescent white (Sigma-Aldrich, St. Louis, MO, USA), and mycelial nuclei were visualised with 20 μg/mL DAPI and 20 μg/mL CFW. To assess the sporulation capacities of the WT and mutant strains, WT, and mutant strains were incubated on PDA plates at 45 °C for 8 days and 37 °C for 13 days. The hyphae and spores were collected with 5 mL of sterilised distilled water, then filtrated through a sterile funnel (containing 4 layers of lens paper), followed by centrifuge at 4 °C 12,000 r/min, for 2 min. The bottom spores were harvested and washed with 1 mL of sterile distilled water and then used for the spore solutions. The spores were counted with a haemocytometer (1/400 m^2^, 25 × 16) (the number of spores = the number of spores in small cells/n × 400 × 10^4^ × dilution factor). Ten μL of 1 × 10^8^/mL spore solution of WT and mutant strains were added in 1 mL YG (5 g/L yeast extract, 20 g/L glucose) liquid medium at 45 °C and 37 °C, and incubated at 180 r/min. The evaluation of the spore germination rate was carried out every two hours (spore germination rate = germinated spore number/total number of spores).

The fungal strains were stained with calcofluor-white (CFW, Sigma-Aldrich, St. Louis, MO, USA), a fluorescent dye that naturally binds to cellulose and chitin. The hyphae were collected using a sterilised coverslip. The fungal strains were stained with 20 μg/mL CFW and then observed under an inverted fluorescence microscope (Nikon, Tokyo, Japan).

### 2.5. Metabolic analysis

Fungal strains *T. dupontii* were cultured on PDA for 7 days at 45 °C, and then 3–4 pieces of 5–6 mm mycelial disks were cut off and inoculated into each 500 mL flask containing 250 mL of potato dextrose broth (PDB, potato 200 g/L and glucose 20 g/L) and incubated at 37 °C, respectively, for 7 days on a rotary shaker (180 r/min). The fermentation broths were exhaustively extracted overnight with 250 mL ethyl acetate (1:1 v/v) and the organic layers were concentrated to dryness under reduced pressure. The dried organic residue was dissolved in 1 mL methanol, Then, 200 μL of samples were dissolved in 500 μL chromatographic methanol filtered through 0.22 μm membranes, and analysed by high-performance liquid chromatography-tandem mass spectrometry (HPLC-MS/MS).

HPLC-MS/MS analysis was performed on a Q Extractive Focus HPLC-MS/MS (Thermofisher, Eugene, OR, USA) with a PDA detector and an Obitrap mass detector (Shiseido, 5 μm, 4.6 mm × 250 mm, CAPCELL PAK C18 column) using positive and negative mode electrospray ionisation. The total flow rate was 1 mL/min; mobile phase A was 0.1% formic acid in water; and mobile phase B was 0.1% formic acid in acetonitrile. The column temperature was maintained at 40 °C. The injection volume for the extracts was 10 μL. The liquid chromatography (LC) conditions were manually optimised based on separation patterns with the following gradient: 0–2 min, 10% B; 10 min, 25% B; 30 min, 50% B; 35 min, 90% B; 36 min, 95% B; 40 min, 95% B; 40.1 min, 10% B; and 45 min, 10% B. UV spectra were recorded at 196–400 nm. The data were analysed and processed using Compound Discoverer 3.0 software (Thermofisher) (Tables S4 and S5).

### 2.6. Transcriptional analysis

The WT and mutant strains were cultured in PDB medium at 37 °C and 45 °C, respectively, for 7 days on a rotary shaker (180 r/min). Three treatment groups with three independent biological replicates were used for each sample. The mycelium samples were sequenced by Guangzhou Kidio Biotechnology Co., Ltd. (Guangzhou, China), and the data were obtained using the OmicSmart online platform (https://www.omicsmart.com/) for analysis. Transcript abundances (FPKM) were provided by SeHealth Tech after sequencing and differentially expressed genes were calculated using R software. All genes with *P* value ≤ 0.05 and log2 (fold_change) ≥ 1 were considered significantly differentially expressed.

### 2.7. General experimental procedures for isolation and characterization of PIAs and their novel analogues

Silica gel 60 (Merck, 230− 400 mesh) was used for column chromatography. Column chromatography was performed on 200−300 mesh silica gel (Qingdao Marine Chemical Factory, Qingdao, China). Optical rotations were measured on a Horiba-SEAP-300 spectropolarimeter. UV spectral data were obtained on a Shimadzu-210A double-beam spectrophotometer. IR spectra were recorded on a Bruker-Tensor-27 spectrometer with KBr pellets. NMR experiments were carried out on either a Bruker AV-400 or a DRX-500 spectrometer with TMS as the internal standard. MS were recorded on a VG-Auto-Spec-3000 spectrometer. High-resolution ESIMS (HRESI MS) were measured on a Bruker Bio-TOF III electrospray ionisation mass spectrometer. Compounds on plates were detected by spraying with 15% (w/v) H_2_SO_4_ and heating on a hot plate. The PIA metabolites, talathermophilins A–F (1–6), were isolated as standard samples from wild-type *T. dupontii* YM3-4 according to the modified methods described in the literature (Chu et al. 2010; Guo et al. 2011).

A total of 80 L amount of PDB culture broth of the strain *T. dupontii* YM3-4 was separated by filtration into the mycelia and filtrate. The filtrate was concentrated to 1.0 L and extracted 5 times with equal volumes of ethyl acetate. The EtOAc layer was combined and evaporated under reduced pressure to give a brown gum (20 g). This gum was loaded onto a macroporous resin column and eluted with H_2_O/MeOH with decreasing polarity (0%–25%–50%–75%–100% MeOH) to yield five fractions (A–E) based on TLC behaviour. Fraction D obtained on elution with 50%–90% MeOH/H_2_O, was further subjected to a Sephadex LH-20 gel column eluting with MeOH to yield four subfractions (A–F). Subfraction C was further subjected to a silica gel column eluting with CHCl_3_/MeOH (10:1–5:1) with increasing polarity to obtain 7–8 (2 mg); Subfraction D (63 mg) was further subjected to a silica gel column eluting with CHCl_3_/MeOH (10:1–5:1–2:1) with increasing polarity to obtain **9** (4 mg); Subfraction E (31 mg) was further subjected to a silica gel column eluting with CHCl_3_/MeOH (9:1–4:1) with increasing polarity to obtain **11** (4 mg).

### 2.8. Scanning electron microscopy (SEM)

To observe conidia and mycelium morphology, a sterile cover slide was inserted into a PDA plate 2 cm from the central point, and WT and mutation were inoculated at the central point. Samples cultured for 6  days were collected and prepared for scanning by soaking them with 4% glutaraldehyde (Sigma-Aldrich, St. Louis, MO, USA) for 20 min. washed twice with sterile distilled water for 15 min each time, and then rinsed and dehydrated with ethanol of different gradients (50%, 70%, 80%, 90%, and 100%) for 15 min each time. Then soak in anhydrous ethanol:isoamyl acetate (1:1) for 15 min each time, and then discard the solution and add isoamyl acetate into the sample. After critical-point drying using a model CPD-030 device (Bal-Tec AG, Balzers, Liechtenstein), the samples were coated with gold using a model SCD 005 sputter coater (Bal-Tec) and observed by scanning electron microscopy (Quanta-200, FEI, Hillsboro, OR, USA).

### 2.9. Transmission electron microscopy (TEM)

The ultrastructural features of fungal WT and mutant cultures were examined using transmission electron microscopy. Fresh mycelia were cultured in PDB for 24 h at 45 °C and 37 °C, respectively to remove any medium, and then were incubated with 2.5% glutaraldehyde (Sigma) in phosphate buffer (pH 7.4) overnight at 4 °C. Conidia were cultured in PDA at 45 °C for 7 days and at 37 °C for 14 days, respectively. The samples were pretreated in 4% paraformaldehyde, then processed with 2.5% glutaraldehyde (Sigma) in phosphate buffer (pH 7.4) overnight at 4 °C and post-fixation in 1% OsO_4_, the samples were dehydrated in a graded ethanol series, embedded in Spurr resin, and stained with 2% uranyl acetate and Reynold’s lead solution. The samples were examined under an H-7650 transmission electron microscope (Hitachi) (Chen et al. 2023; Yang et al. 2023).

### 2.10. Iron measurement

Iron levels (Fe^2+^ and Fe^3+^) in fungal strains were evaluated with Iron assay kit (#I291, Dojindo). In Brief, 150 mg mycelia were collected and washed with cold PBS three times. The fungal mycelia were re-suspended in iron assay buffer and homogenised using the homogeniser sitting on ice. Then, the mixtures were centrifuged at 12,000 r/min for 10 min at 4 °C, and 100 μL supernatants were collected for detection. Next iron assay buffer/reducer was added to the collected supernatant, mixed, and incubated. The incubated solution with an iron probe for 1 h in the dark was immediately measured on a microplate reader at OD = 593 nm.

### 2.11. Data analysis

Data from three biological repeated experiments were expressed as means ± SD, which were analysed by one-way analysis of variance followed by Tukey’s multiple comparison test, with *P* values < 0.05: *; *P* values < 0.01: **; *P* values < 0.001: ***, considered statistically significant. All statistical analyses were conducted using GraphPad Prism ver. 8.2.1 for Windows (GraphPad Software, San Diego, CA, USA).

### 2.12. Accession numbers

*dupontii P450*S: GME4769_g; *T. dupontii P450*L: GME4767_g; Transcriptomic data: PRJNA977981.

## 3. Results

### 3.1. *Unique gene fusion and diversification of* P450 *paralogs in the PIA cluster in* T. dupontii

Our previous study suggested that five key biosynthetic genes in the *PIA* gene cluster were most up-regulated in *T. dupontii* at a minimum growth temperature of 37 °C compared to the optimal growth temperature of 45–50 °C (Chen et al. 2023). Detailed analysis in this study revealed that two *P450* paralogs [GME4769_g designated as P450S (1,308 bp) and GME4767_g as P450L (2,586 bp) according to their lengths] were remarkably up-regulated with 57-fold and 48-fold higher at 37 °C than at 45 °C, respectively (Figure 1b). InterProScan analysis revealed that both *P450L* and *P450S* were members of the cytochrome P450 (IPR001128) family. Surprisingly, further bioinformatics analysis revealed that no homologues of the large P450L in *T. dupontii* were found in all the known *PIA* gene clusters (Figure 1c), including *not* cluster from a marine *Aspergillus* species, *ech* cluster from common *A. ruber*, and *has* cluster from human pathogen *A. fumigates*.

Using the P450L and P450S protein sequences in *T. dupontii* as queries, we conducted reciprocal BLASTP against the NCBI Fungal database (taxid: 4751). The best BLAST hits for each protein were recorded and used for further phylogenetic analysis. In the gene tree (NJ tree), P450S and P450L fell into separate clades, which were designated as P450S and P450L clades, respectively (Figure 1d). All the proteins in the P450S clade contained a single CYP67-like conserved domain with a sequence coverage ranging from 96% to 100%. However, the first half of the P450L sequence is well aligned with the proteins containing a CYP64-like conserved domain, while the second half is well aligned with the proteins possessing a FAD-binding domain, such as oxidoreductase. This implies that P450L should be a protein with multiple distinct functions. To eliminate potential errors caused by the gene structure prediction process, we assembled the transcripts from RNA-seq datasets using Trinity and carried out gene predictions using Transdecoder (Grabherr et al. 2011). Program to Assemble Spliced Alignments (PASA) was applied to perform alternatively spliced isoforms for the genes (Haas et al. 2008). We integrated PASA into our gene prediction pipeline to identify alternative splice variants. Two gene prediction pipelines also supported that P450L is a single protein possessing three distinct domains (Figure 1d). We conducted a homology search for P450L using the NCBI database and found that the first half of P450L (24–510 aa) encoded a CYP64-like domain, and the second half (513–894 aa) encoded a FAD-binding domain-containing oxidoreductase. Coding sequences alignment analysis of P450L, P450 from *Penicillium italicum* (PiP450, with 53.0% coverage and 54.10% identity) and FAD-binding domain-containing oxidoreductase from *Aspergillus sclerotiicarbonarius* (*AsFOx* with 42.0% coverage and 51.6% identity) indicated that *P450L* gene had 273 bp less than the sum of two separate genes *PiP450* and *AsFOx*. Interestingly, the first half sequence of gene *P450L* encoding the CYP64-like domain lost 64 bp in the last exon and the second half encoding the FAD-binding domain-containing oxidoreductase lost 209 bp in the first exon, leading to the fusion of these two domain encoding sequences to form the gene *P450L*. To further confirm this result, we extracted genome region (49,998 bp) containing gene *P450L* and analysed it with the FGENESH (v.2.6) program on the Softberry website (http://www.softberry.com/) and 2ndFind (https://biosyn.nih.go.jp/2ndfind/). Fgenesh and 2ndFind also recognised *P450L* as a complete gene encoding a single protein composed of 1,010 amino acids and 1,114 amino acids, respectively. When expanding the query to include all fungal protein sequences in MycoCosm (Grigoriev et al. 2014), we did not find another case that a P450 composed of a CYP64-like domain fused with a FAD-binding domain-containing oxidoreductase.

### 3.2. *The mutants with the disruption of* P450S/P450L *were constructed via a CRISPR/Cas9 gene editing system and confirmed via sequencing and metabolic analysis*

**Figure 2.**
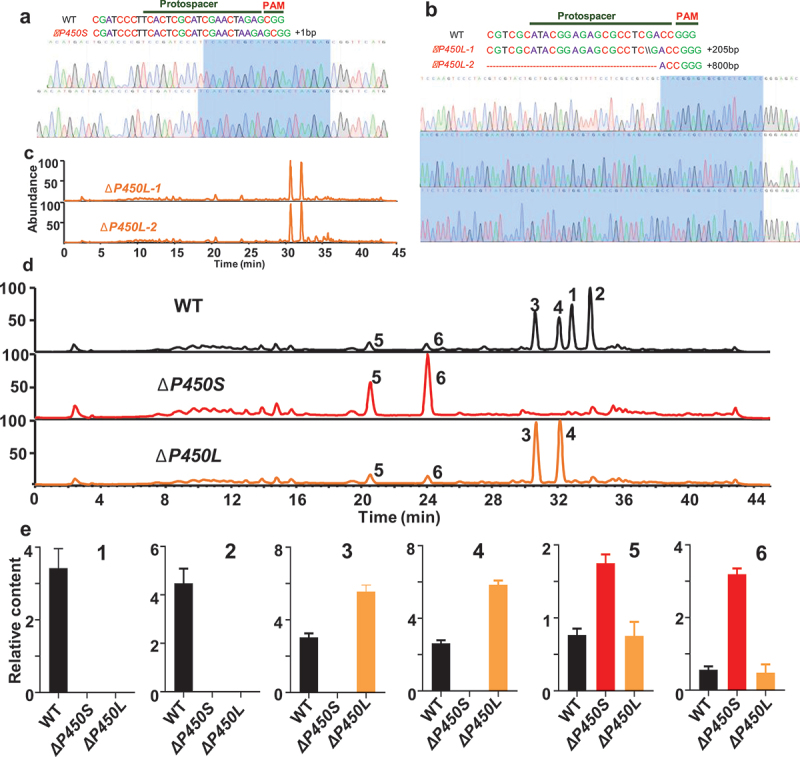
(a–b) Sequence analysis of disruption mutants *ΔP450S* and *ΔP450L* constructed with a CRISPR/Cas9 gene editing system for *Thermomyces dupontii*. (c) Metabolic profiles of two *ΔP450L* mutants and wild-type (WT) at 37 °C. (d) Comparison of metabolic profiles between two *T. dupontii* mutants *ΔP450S* and *ΔP450L*, and WT at 37 °C indicated that the disruption of *ΔP450S* led to the loss of the pyran prenylated indole alkaloids (PPIAs) (1–4) and the disruption of *ΔP450L* resulted in the loss of PIAs (1–2). (e) Comparison of the contents of the major prenylated indole alkaloids (PIAs) (1–6) accumulated in two mutants *ΔP450S* and *ΔP450L*, and WT at 37 °C.

The CRISPR/Cas9 gene editing system was successfully adapted for genome editing in *T*. *dupontii* using an *in vivo* sgRNA expression under the control of the tRNA^Gly^ self-processing system and a fungal-optimised Cas9 nuclease from the bacterium *Streptococcus pyogenes* (SpCas9) (Huang et al. 2020). The CRISPR/Cas9 gene editing system was applied to construct the mutants with disruption of *P450S* and *P450L*, respectively. PCR and sequence analysis suggested that one mutant *ΔP450S* and two mutants *ΔP450L*-1 and *ΔP450L*-2, were finally achieved (Figure 2a,b). The lengths of genes *P450S* and *P450L* are 1,479 bp and 3,497 bp, respectively. The mutant Δ*P450S* had one more base A inserted at the 210 bp of gene *P450S*, which resulted in mutation from the 70th amino acid of the corresponding protein. Mutants Δ*P450L-1* and Δ*P450L-2* had one extra fragment of 205 bp and about 800 bp inserted at the 355 bp of gene *P450L*, respectively, which caused mutation from the 110th amino acid of the corresponding proteins. All the mutants and WT were cultured on 250 mL PDB medium in a 500 mL flask and the cultural broths were extracted with the same volume of ethyl acetate. Then HPLC-MS/DAD analysis was applied to perform metabolic profiles of the extracts. Because both *ΔP450L*-1 and *ΔP450L*-2 shared the same metabolic profiles (Figure 2c), thus one mutant was chosen for the next experiments.

### 3.3. *Metabolic analysis indicated that* P450S *and* P450L *played distinct roles in bolstering simple PIA to more stable complex PPIAs*

The HPLC-MS/MS analysis revealed that *ΔP450S* and *ΔP450L* displayed distinct metabolic profiles from WT at 37 °C (Figure 2d). Metabolites 3–4 could not be detected in *ΔP450S*. Instead, metabolites 5–6 were dramatically accumulated in *ΔP450S* (Figure 2d–e). These results suggested that *ΔP450S* was also involved in the biosynthesis of 3–4. Thus, *P450S* was responsible for the hydroxylation at the phenyl ring of 5–6 to form the pyrano ring. Notably, metabolites 1–2 disappeared in both *ΔP450S* and *ΔP450L* while 3–6 existed in *ΔP450L* (Figure 2c–e), suggesting that both *P450S* and *P450L* were responsible for the biosynthesis of 1–2 and *P450L* was responsible for the dehydrogenation of 3–4 to yield 1–2. The chemical function of gene *P450L* in dehydrogenation was consistent with the chemical function of the CYP64-like domain gene *echP450* of *A. ruber* in the introduction of double bond (Nies and Li 2021) and previous studies that FAD-domain containing oxidoreductase could introduce a double bond because FAD was assigned as a proton-receptor (Rosberg-Cody et al. 2011; Iijima et al. 2019; Lee et al. 2021).

### 3.4. P450L *was responsible for the formation of simple PIA-derived iron chelators*

**Figure 3.**
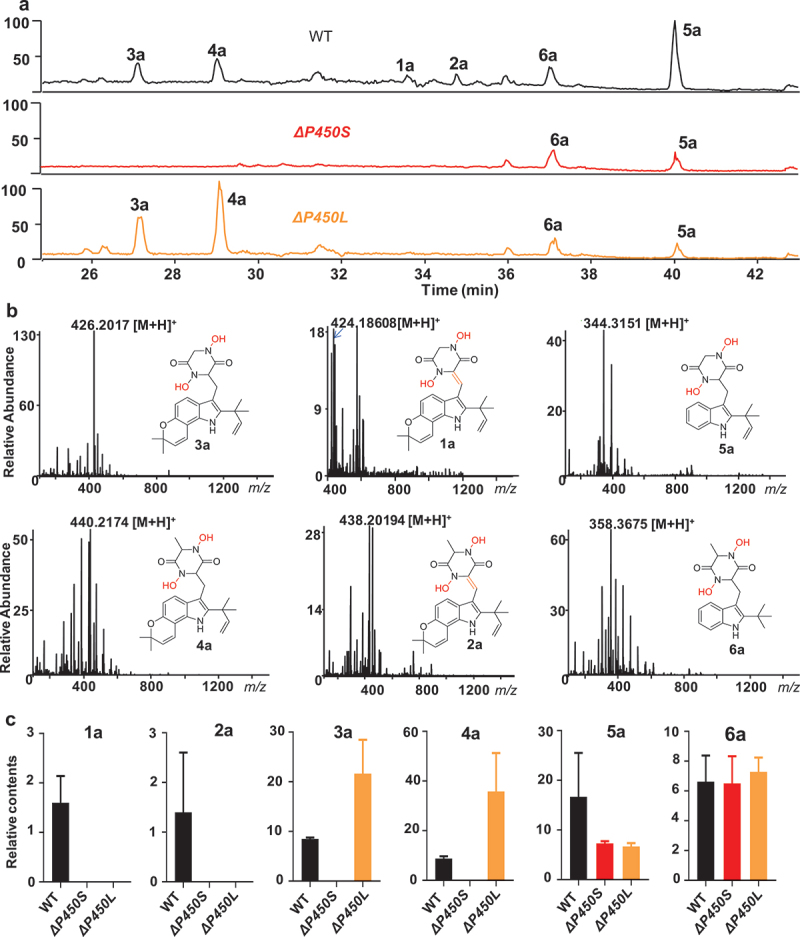
(a) HPLC-MS analysis of the hydroxyl derivatives 1a–6a of prenylated indole alkaloids (PIAs) 1–6 in mutants *ΔP450S* and *ΔP450L* and WT. Full-scan+ mode spectra were acquired over a scan range of *m/z* 100–1,500. All the iron chelators 1a–6a that possessed two hydroxamate moieties for chelating iron were detected in the WT. Metabolites 5a–6a in *ΔP450S* and metabolites 3a–6a in *ΔP450L*. (b) The diagnostic [M+H]^+^ peaks in the high-resolution ESI MS for 1a–6a. (c) Comparison of the contents of the major iron chelators 1a–6a in two mutants *ΔP450S* and *ΔP450L*, and WT at 37 °C.

Previous studies have suggested that HPLC-MS analysis was applied to detect the iron-chelating metabolites derived from PIAs in the fungal cultural broths, such as astechrome from *A. terreus* and hexadehydroastechrome from *A. fumigates* (Arai et al. 1981; Yin et al. 2013; Bok et al. 2015). As expected, the HPLC-MS profile of *T. dupontii* WT at 37 °C displayed the diagnostic molecular ion peaks for all the iron chelators (1a–6a) derived from 1–6 with two hydroxamate cores (Figure 3a,b). Further solid evidence came from the metabolic profiles that a known iron chelator, 11,14-dihydroxylneoechinulin E, derived from 5, was highly enriched in WT while was extremely dropped in two mutants *ΔP450S* and *ΔP450L* (Figure S1).

Notably, 5a–6a were also found in both mutants *ΔP450S* and *ΔP450L* (Figure 3a), but the content level of 5a significantly decreased in *ΔP450S* and *ΔP450L*, suggesting that either *P450S* or *P450L* could transform simple PIAs 5–6 to their corresponding iron chelators 5a–6a (Figure 3c). The chemical functions of *P450S* and *P450L* in the formation of two hydroxamate cores of iron chelators 5a–6a were consistent with the chemical functions of *P450* analogues in the *PIA* gene clusters in other fungal species in the formation of these two hydroxamate cores.

### 3.5. P450L *was involved in the formation of the third hydroxamate core in iron chelator derived from novel PPIA*

**Figure 4.**
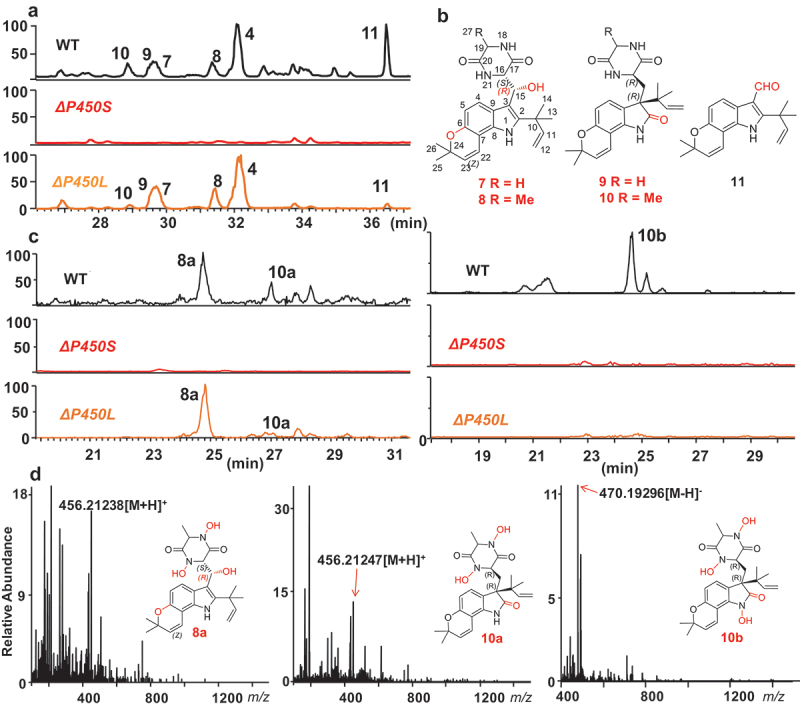
(a) Detailed comparison of metabolic profiles between two *Thermomyces dupontii* mutants *ΔP450S* and *ΔP450L*, and WT at 37 °C indicated that some extra prenylated indole alkaloids (PIAs) (7–11) were lost in *ΔP450S*. (b) The structures of five target PIAs (7–11), including four new ones (7–10) isolated and characterized in this study. (c) HPLC-MS analysis of the hydroxyl derivatives of PIAs 7–10. Full-scan+ and full-scan–mode spectra were acquired over a scan range of *m/z* 100–1,500. Two iron chelators 8a and 10a that possessed two hydroxamate moieties were detected in the WT and *ΔP450L*. The iron chelator 10b which harbored three hydroxamate moieties for chelating iron was only found in WT. (d) The diagnostic [M+H]^+^ peaks in the high-resolution ESI MS for 8a and 10a and the diagnostic [M-H]^–^ peak in the HRESI MS for 10b.

Detailed comparison of metabolic profiles of the two mutants and WT revealed that disruption of *P450S* resulted in the absence of several [M+H]^+^ peaks at *m/z* 410, 424, and 296 in the positive ESIMS in the fungus at 37 °C (Figure 4a). These target metabolites seemed to possess more complex oxidation patterns than all the known talathermophilins (1–6). To characterise these specific target metabolites, 80 L of PDA cultural broth of *T. dupontii* WT strain was fermented at a minimum growth temperature of 37 °C, and a chemical investigation was carried out. Three new metabolites (7–9) (Figure 4b), together with seven known PIAs (1–6 and 11), were achieved and their structures were determined based on HRESI MS and NMR data (Tables S1 and S2).

Compound 7 was determined to have a molecular formula of C_23_H_27_N_3_O_4_ based on a [M+H]^+^ peak at *m/z* 410.2084 in the HRESIMS, which was confirmed by NMR spectrometric data (Tables S1–S2). The ^1^H NMR, ^13^C NMR, and DEPT spectra of 7 (Figures S6 and S10) displayed the diagnostic signals for a tetrasubstituted indole core, a diketopiperazine moiety, and two isoprenyl groups. Comparison of the ^1^H and ^13^C NMR data of 7 with those of the previously reported key intermediate talathermophilin C (3) demonstrated that these two compounds shared the same skeleton except that 7 had one more hydroxyl group, which was confirmed by its ESIMS. One methylene group (CH_2_-15, *δ*_C_ 31.0 t; *δ*_H_ 3.40 dd, *J* = 14.6, 4.3 Hz; 3.38 dd, *J* = 14.6, 8.4 Hz) in 3 was replaced by a hydroxylmethine group (*δ*_*C*_ 70.69 d; *δ*_H_ 5.55 d, *J* = 7.4 Hz) in 7. The strong^1^H-^13^C correlations of H-15 with C-2 (*δ*_*C*_ 143.18 s), C-3 (*δ*_*C*_ 110.05 s), C-9 (*δ*_*C*_ 123.33 s), C-16 (*δ*_*C*_ 61.69 d), and C-17 (*δ*_*C*_ 170.43 s) in the HMBC spectrum of 7 confirmed the oxygenated pattern at C-15 in 7 as shown in Figure 4. Unambiguous assignments of the NMR data of 7 were achieved through 2D NMR (^1^H-^1^H COSY, HSQC, HMBC, and ROESY) experiments. Because the NOESY spectrum of 7 could not be used for the characterisation of the configuration of the OH at C-15, we tried to obtain the ^1^H NMR analysis of the corresponding 15-OH (R)- and (S)-MTPA esters of 7. However, we failed to achieve the R and S-MTPA esters of 7. So, we turned to apply the ECD spectrum. The experimental ECD spectrum (Figure S2) was identical to the calculated ECD spectrum of 7 with R-configuration at C-15 and S-configuration at C-16, suggesting the structure of 7 as described in Figure 4b.

Compound 8 was assigned to a molecular formula of C_24_H_29_N_3_O_4_ based on a [M+H]^+^ peak at *m/z* 424.2232, which was confirmed by NMR spectrometric data (Figures S11–S16), indicating that 8 had one more CH_2_ unit than 7. The UV and IR spectra of 8 were almost identical to those of 7. Furthermore, the^1^H and^13^C NMR data of 8 were similar to those of 7 except that the signals for one CH_2_ (*δ*_*C*_ 45.78 t; *δ*_H_ 3.62 d, *J* = 2.8 Hz) in 7 were replaced by the signals for one CH (*δ*_*C*_ 52.05 d; *δ*_H_ 3.92 q, *J* = 6.9) and one CH_3_ (*δ*_*C*_ 20.29 q; *δ*_H_ 5.55 d, *J* = 7.4 Hz) in 8. Unambiguous assignments of the NMR data of 8 were achieved through 2D NMR (^1^H−^1^H COSY, HSQC, HMBC, and ROESY) experiments, and 8 was characterised as the methyl-19 derivative of 7. The structures of 7 and 8 are closely related to the previously reported key intermediates, talathermophilins C–D (3–4), which were originally derived from the condensation of tryptophan with glycine or alanine.

Compound 9 shared the same molecular formula of C_23_H_27_N_3_O_4_ as compound 7 based on a [M+H]^+^ peak at *m/z* 410.2086 in the HRESIMS, which was confirmed by NMR spectrometric data (Figures S17–S21). However, the^1^ H NMR, ^13^C NMR, and DEPT spectra of 9 were different from those of 7. Detailed analysis suggested that the indole core in 7 might be replaced by an indolin-2-one in 9 based on the diagnostic signals (*δ*_C_ 180.75, C and 55.41, C) in the^13^C NMR spectrum of 9. Further comparison of the NMR data of metabolites 9 and 3 revealed that both compounds shared similar CH_2_-15. All the protons were assigned to the corresponding carbons according to the HSQC spectrum of 9. The 1H−^13^C long-distance correlations of the quaternary carbon (*δ*_C_ 55. 41) with NH-1 (*δ*_H_ 10.79), H-4 (*δ*_H_ 6.84), H-11 (*δ*_H_ 5.99), H_2_-12 (*δ*_H_ 5.06 and 4.96), H_3_-13 (*δ*_H_ 0.99) and H_3_-14 (*δ*_H_ 0.92) indicated the presence of the prenyl unit at C-3 in 9, instead of at C-2 in 7. The experimental ECD spectrum and the calculated ECD spectrum of 9 (Figure S2) suggested that the stereochemistry of C–3 and C–16 were both *R* configurations.

**Figure 5.**
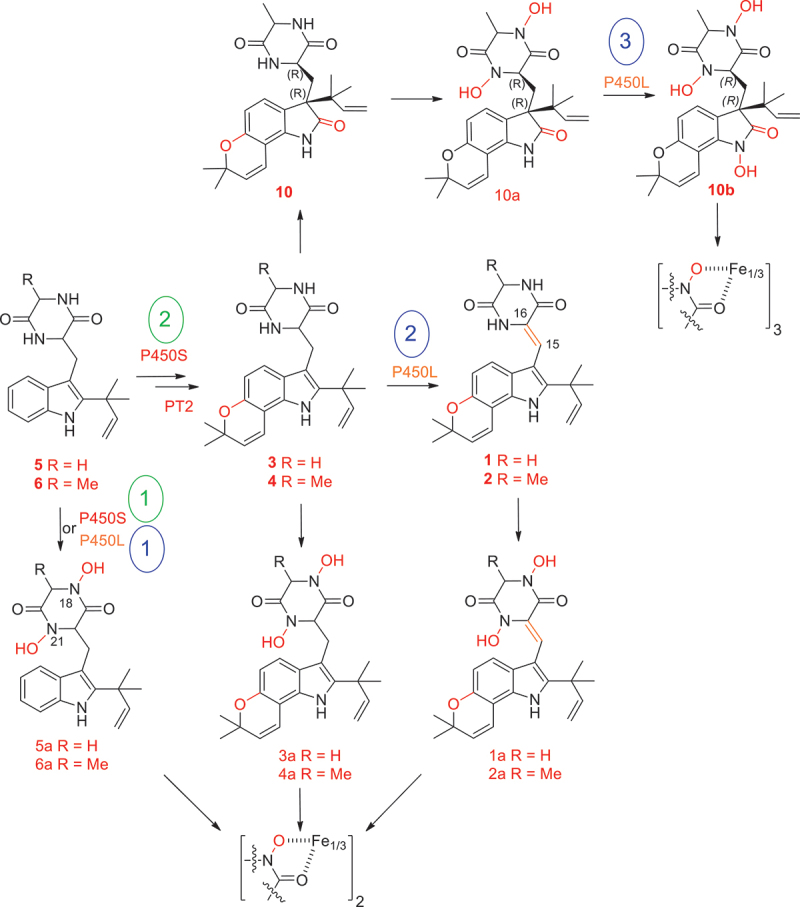
The multi-functions of genes *P450S* and *P450L* in biosynthetic pathways for two simple prenylated indole alkaloids (PIAs) and five complex pyran prenylated indole alkaloids (PPIAs) and their associated iron chelators. Gene *P450S* was involved in the formation of two hydroxamate moieties in iron chelators 5a–6a (1, green) and the pyran ring in PPIAs 1–4 (2, green). Gene *P450L* was responsible for the formation of two hydroxamate moieties in iron chelators 5a–6a (1, blue), the double bond at C15–C16 in PPIAs 1–2 (2, blue), and the third hydroxamate core in iron chelator 10b derived from novel PPIA 10 (3, blue).

Thus, the compounds 7–9 were three new derivatives of talathermophilins C–D. Among them, 7–8 were two new 15-hydroxyl talathermophilins C–D (3–4), and compound 9 was a new 2-ketone derivative of talathermophilin C (3). There might be a new 2-ketone derivative (10) of talathermophilin D (4), namely, the methyl analogue of 9 in WT (Figure 4a). Unfortunately, we failed to obtain metabolite 10 most likely due to its very low trace amount in the fungus. Moreover, a broken fragment metabolite (11) derived from the oxygenation of talathermophilins A–B (1–2) was also characterised as described in Figure 4b according to their HRESI MS, 1D, and 2D NMR spectra (Figures S22–S26). As expected, *T. dupontii* WT at 37 °C displayed diagnostic molecular ion peaks for iron chelators 8a and 10a with two hydroxamate cores derived from 8 and 10 (Figure 4c,d). However, none of the iron chelators 7a and 9a with two hydroxamate cores derived from 7 and 9 were detected in *T. dupontii* WT at 37 °C (Figure 4c). These results suggested that PPIAs derived from alanine, 8 and 10, might be more easily transformed to iron chelators than those derived from glycine, 7 and 9, which was consistent with the fact that iron chelators in fungi were commonly derived from alanine (Yin et al. 2013). Due to the emerging ketone at C-2 in 10a, we supposed that there might be one more place for the third hydroxamate core in 10a. Indeed, in the metabolic profile of WT at 37 °C, there existed a diagnostic molecular ion peak for 10b (Figure 4c,d). Notably, the disruption of gene *P450L* caused a loss of 10b in Δ*P450L* (Figure 4c), suggesting that *P450L* was involved in the formation of the third hydroxamate core in 10b (Figure 5). These results might explain why metabolite 10 was in a very low trace amount because metabolite 10 might be quickly transformed to 10a and 10b.

### 3.6. *Both* P450S *and* P450L *facilitated pigments, growth, conidial formation, and germination during temperature reduction*

**Figure 6.**
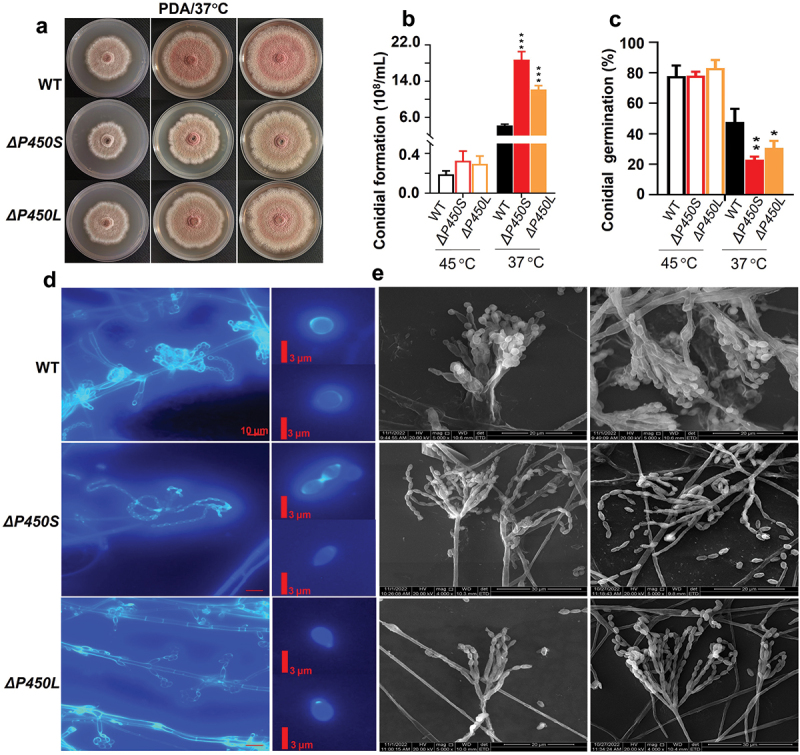
The multi-functions of genes *P450S* and *P450L* in bolstering fungal pigments, conidiophores, and cell walls. (a) Comparison of pigment profiles and growths between two *Thermomyces dupontii* mutants *ΔP450S* and *ΔP450L*, and WT on PDA at 37 °C. (b–c) Comparison of conidial formation (b) and germination rates (c) between two *T. dupontii* mutants *ΔP450S* and *ΔP450L*, and WT on PDA at both temperatures 37 °C and 45 °C. (d) Comparison of mycelia and conidia stained with fluorescent dye calcofluor white (CFW) that naturally binds to cellulose and chitin cell, between two *T. dupontii* mutants *ΔP450S* and *ΔP450L*, and WT on PDA at 37 °C. (e) SEM analysis of conidiophores of two *T. dupontii* mutants *ΔP450S* and *ΔP450L*, and WT on PDA at 37 °C.

The degree of conjugation in a structure is directly proportional to its colour intensity. Figure 6a demonstrated the effects of genes *P450S* and *P450L* on fungal colony pigments which were associated with the metabolites modified by P450S and P450L. We observed that the mutants *ΔP450S* and *ΔP450L* displayed fewer pigments than WT on PDA at 37 °C (Figure 6a). Among them, *ΔP450S* showed fewer pigments than *ΔP450L* (Figure 6a), consistent with the above result that *P450S* was involved in converting simple PIAs (5–6) into complex PPIAs (3–4) while *P450L* in enhancing the linkage between the complex pyranoindole ring and the diketopiperazine in PPIAs (1–2) (Figure 5). These findings suggest that metabolites 1–2 accumulated in WT could give rise to the strongest pigments among the three strains, and metabolites 3–4 accumulated in *ΔP450L* could give rise to stronger pigments than 5–6 accumulated in *ΔP450S*.

Further phenotype analysis revealed that both two mutants displayed retarded growth compared to WT on two other tested media YMA and TYGA. The fungal growth retardation became much worse at 37 °C than at 45 °C. Among them, *ΔP450S* displayed much more growth retardation than *ΔP450L* (Figure S3). Additionally, both *ΔP450S* and *ΔP450L* displayed largely increased conidial formations at a minimal growth temperature of 37 °C with 4.4-fold and 2.9-fold higher than WT, respectively (Figure 6b). However, both two mutants exhibited strongly decreased conidial germination rates compared to WT with decreased rates at 51.7% and 35.6% at 8 h, respectively (Figure 6c).

### 3.7. P450S *and* P450L *were involved in bolstering conidiophores and cell walls*

Staining two mutants and WT with CFW that naturally binds to cellulose and chitin, we found that WT at 37 °C displayed clustered penicillins, while no such structures were observed in both *ΔP450S* and *ΔP450L* at 37 °C (Figure 6d). Importantly, the WT conidia were globose in shape, while *ΔP450S* and *ΔP450L* conidia were abnormally elliptical. In particular, *ΔP450S* displayed more fused immature conidia than *ΔP450L* (Figure 6d). Further SEM analysis revealed that both *ΔP450S* and *ΔP450L* displayed distinct penicillin structures from WT (Figure 6e). Detailed analysis revealed that the conidiophores, metullae, and stigmata structures of WT were stouter than those of two mutants *ΔP450S* and *ΔP450L*. The metullae structures on one conidiophore in two mutants *ΔP450S* and *ΔP450L* were all separate, while those in WT were bundled up to form one broad and thick metullae structure (Figure 6e). Moreover, *ΔP450S* displayed more abnormal penicillins and conidia than *ΔP450L*. These observations indicated that the WT conidiophores were bound into bundles at a relatively low temperature of 37 °C, and both *P450S* and *P450L* played key roles in binding the separate metullae structures into stout penicillin for conidiophores at low temperatures.

**Figure 7.**
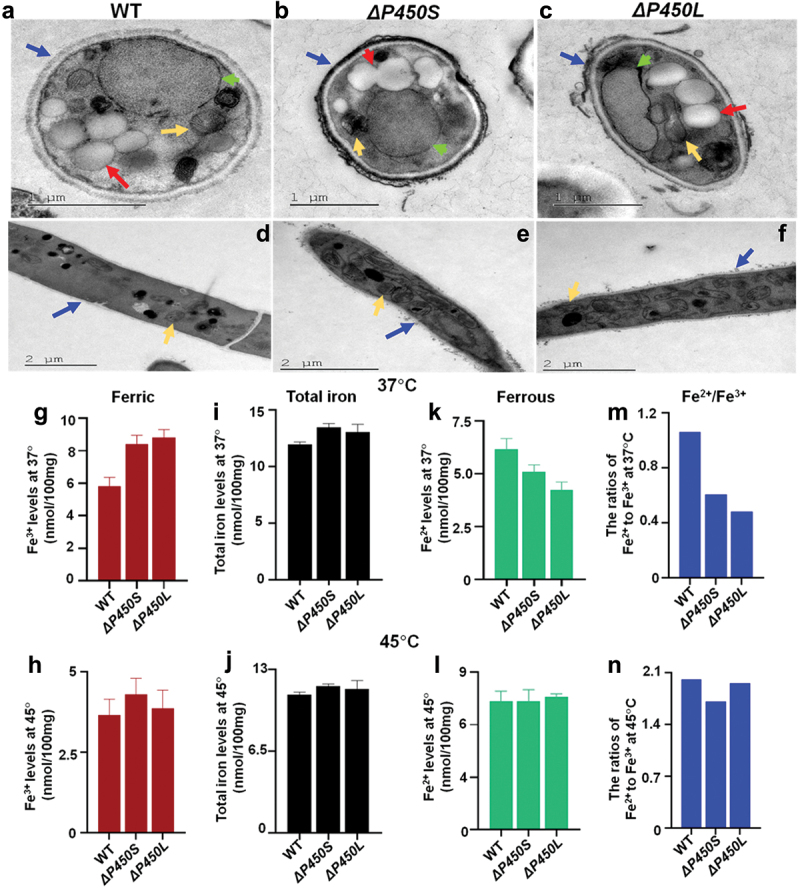
The effects of genes *P450S* and *P450L* on maintaining organelles in conidia and mycelia via regulating iron ratios. (a–f) TEM analysis of conidia (a–c) and mycelia (d–f) of two *Thermomyces dupontii* mutants *ΔP450S* and *ΔP450L*, and WT at 37 °C. The blue arrow refers to the cell wall; the yellow arrow refers to mitochondrion; the red arrow refers to lipid droplets; the green arrow refers to vacuoles. (g–n) Comparison of iron levels and ratios in two mutants *ΔP450S* and *ΔP450L* and WT at 37 °C and 45 °C, respectively. (g–h) Comparison of the levels of Fe^3+^ in two mutants and WT at 37 °C and 45 °C, respectively. (i–j) Comparison of the levels of total free iron ions in two mutants and WT at 37 °C and 45 °C, respectively. (k–l) Comparison of the levels of Fe^2+^ in two mutants and WT at 37 °C and 45 °C, respectively. (m–n) Comparison of the Fe^2+^/Fe^3+^ ratio in WT two mutants at 37 °C and 45 °C, respectively.

TEM analysis was carried out to compare the cell walls of two mutants and WT. Both *ΔP450S* and *ΔP450L* displayed distinct cell walls with mucus protein protrusions, compared to WT (Figure 7a–c). The phenomenon was even more pronounced in the conidia, as both *ΔP450S* and *ΔP450L* had dark protrusions outside the conidial cell walls while WT did not. Moreover, the extra protrusions in *ΔP450S* were much denser and larger than those in *ΔP450L*, suggesting that the cell walls in *ΔP450S* were more abnormal than those in *ΔP450L*. These observations were consistent with the differences in conidiophore and conidial formation between *ΔP450S* and *ΔP450L*, aligning with the fact that P450S is upstream of P450L in the biosynthetic pathway for PPIAs (Figure 5).

### 3.8. P450S *and* P450L *played distinct roles in lipid droplets and vacuoles in conidia, and similar influences in mitochondria contents in mycelia via controlling iron metabolisms*

It was observed that *ΔP450S* displayed abnormally fused lipid droplets in conidia while WT and *ΔP450L* did not (Figure 7a–c). Meanwhile, vacuoles in conidia were significantly shrunk in two mutants *ΔP450S* and *ΔP450L*, compared to those in WT (Figure 7a–c). In particular, the vacuole in *ΔP450L* conidia was deformed. Furthermore, both *ΔP450S* and *ΔP450L* at 37 °C displayed distinct mitochondrial shapes and contents in both conidia and mycelia from WT (Figure 7a–f). Remarkably, both *ΔP450S* and *ΔP450L* displayed strongly increased mitochondrial contents in the mycelia, compared to WT.

The levels of Fe^3+^ and total free iron ions in two mutants and WT at 37 °C and 45 °C were evaluated in mycelia which produce metabolites since iron chelators were involved in sequestering Fe^3+^ (Figure 7g–j). Both mutants *ΔP450S* and *ΔP450L* displayed elevated levels of Fe^3+^ and total free iron ions at 37 °C and 45 °C, compared with WT, aligning with the loss of PPIAs-derived iron chelators in both mutants *ΔP450S* and *ΔP450L*. The increases in the levels of Fe^3+^ and total free iron ions between two mutants and WT at 37 °C were higher than those at 45 °C, consistent with the increased transcriptional levels of both *P450S* and *P450L* at 37 °C *vs.* at 45 °C. Further analysis revealed that the levels of Fe^2+^ in both two mutants *ΔP450S* and *ΔP450L* were lower than those in WT at 37 °C but not at 45 °C (Figure 7k–l), indicating that the two *P450*-mediated PPIAs and their corresponding iron chelators were involved in increasing the levels of Fe^2+^ at cold stress. Remarkably, both two mutants *ΔP450S* and *ΔP450L* exhibited strongly decreased ratios of Fe^2+^/Fe^3+^ compared to WT at 37 °C, while only *ΔP450S* showed slightly decreased ratios of Fe^2+^/Fe^3+^ at 45 °C (Figure 7m–n).

## 4. Discussion

The mechanisms of *P450* genes involved in fungal adaption to temperature changes remain largely unclear. In this context, we discovered that the dominant thermophilic fungus *T. dupontii* had the duplication and subsequent diversification of *P450* genes in the *PIA* gene cluster responsible for PPIAs. Interestingly, one *P450* paralog (*P450L*) is almost twice as long as the other one (*P450S*) in sequence lengths. P450S was assigned to contain a single CYP67-like domain, while P450L is a unique large protein harbouring a CYP64-like domain fused with a FAD-binding domain-containing oxidoreductase. The CYP64-like domain and the FAD-binding domain-containing oxidoreductase were separately encoded by two independent genes in all the other fungi while only in one protein P450L of *T. dupontii*. Thus, the *PIA* gene cluster in *T. dupontii* practically encodes two CYP-like domains and one FAD-binding domain-containing oxidoreductase. Among the known *PIA* clusters from other fungi, only the *not* cluster from a marine *Aspergillus* species MF297-2 encodes two CYP-like domains and one FAD-binding domain containing oxidoreductase (Ding et al. 2010). The *ech* cluster from mesophilic fungus *A. ruber* encodes only one CYP-like domain (Nies and Li 2021) and the *has* cluster from human pathogen *A. fumigates* encodes one CYP-like domain and one FAD-binding domain-containing oxidoreductase (Yin et al. 2013).

The PIAs via the *ech* cluster from common mesophilic fungus *A. ruber* (Nies and Li 2021) and the *has* cluster from human pathogen *A. fumigates* belonged to simple PIAs without the pyran ring (Arai et al. 1981; Yin et al. 2013). The PPIA metabolites from *T. dupontii* shared the same pyrano ring as PPIAs from the marine *Aspergillus* species MF297-2 (Ding et al. 2010), consistent with that the two different strains share similar domains, including two CYP-like domains and one FAD-binding domain-containing oxidoreductase. The fact that thermophilic fungus *T. dupontii* was quite similar to the marine fungus *Aspergillus* species MF297-2 in the PPIA biosynthesis suggests that they might have to deal with quite similar environmental changes. The discrepancy between the PIA clusters from the thermophilic fungus *T. dupontii* and the marine fungus *Aspergillus* species MF297-2 may have arisen from gene fusion in *P450L* in *T. dupontii* (Leonard and Richards 2012). Most previous studies suggest that gene fusions are clinically relevant to some types of solid tumours and about 16% of cancers have a gene fusion as one of their drivers (Mertens et al. 2015; Glenfield and Innan 2021). To date, no studies on the function of such a gene fusion in fungi have been reported. This might suggest that the gene fusion that occurred in *P450L* of *T. dupontii* might facilitate the adaptive ability of the thermophilic fungus to temperature changes.

Our findings indicate that both *P450* genes are responsible for transforming simple PIAs (5–6) to iron chelators (5a–6a) by forming two hydroxamate cores, which chelate two-thirds of ferric ions (Figure 5). Meanwhile, *P450S* and *P450L* play distinct roles in converting simple PIAs (5–6) into much more stable PPIAs (1–4), which can be further oxidised to novel PPIAs (7–10) with more oxidations. Specifically, *P450S* is responsible for converting simple PIAs (5–6) to PPIAs (3–4), while *P450L* enhances the link between the complex pyranoindole ring and the diketopiperazine in PPIAs (1–2) (Figure 5). Importantly, the fusion gene *P450L* has the third function in forming the third hydroxamate core in iron chelator 10b derived from 10, which enables the PPIA-derived iron chelator to sequester one whole ferric ion (Figure 5).

It is noteworthy that disruption in either *P450S* or *P450L* resulted in the loss of the supremely stout conidiophores and damaged fungal cell walls. Since P450L was downstream P450S in the biosynthetic pathway for PPIAs, the loss of P450L only caused the loss of the most stable PPIAs (1–2) and their corresponding iron chelators (1a–2a), and the most complex iron chelator 10b in mutant Δ*P450L*. All the results suggested that the bolstered PPIAs (1–2) and iron chelators (1a–2a, and 10a) via P450L were responsible for consolidating fungal conidiophores and cell walls under cold stress.

In addition, the elevated levels of Fe^3+^ and total free iron ions in both Δ*P450S* and Δ*P450L* at relatively low temperatures suggested that PPIA-derived iron chelators play a crucial role in sequestering ferric and controlling total free irons in the fungus in response to cold stress. Interestingly, the significantly reduced Fe^2+^ levels in both mutants Δ*P450S* and Δ*P450L* indicate that both genes *P450S* and *P450L* are also responsible for improving Fe^2+^ levels. Fe^2+^ is stable under anaerobic conditions or at low pHs. Thus, the oxygen-dependent biosynthesis of PPIA and their derived iron chelators via *P450S* and *P450L* are more favourable to Fe^2+^. Another piece of evidence comes from the increased mitochondrial contents in both Δ*P450S* and Δ*P450L* mycelia, as more mitochondrial contents commonly favour more hydrogen protons (H^+^) (Li et al. 2010; Paul et al. 2017). Previous studies have indicated that iron metabolism and mitochondria homoeostasis strongly affected iron-storage vacuoles and energy-storage lipid droplets (Li et al. 2010; Liesa 2022; Aghabi et al. 2023). We also found that the un-normal mycelia with increased mitochondrial contents resulted in Δ*P450S* conidia with un-separated lipid droplets and Δ*P450L* conidia with deformed vacuoles, consistent with the decreased conidial germination rates in both mutants under cold stress.

In our search for siderophore gene clusters within the *T. dupontii* genome using common types of siderophore synthetases from Ascomycota as a query, we found no homologous siderophore gene clusters. However, we unexpectedly discovered a basidiomycete type VI siderophore synthetases (responsible for the synthesis of basidioferrin), which is widely distributed in Basidiomycetes (Brandenburger et al. 2017). Transcriptomic data showed that the genes in basidioferrin-like gene cluster in *T. dupontii* were highly expressed at 45 °C compared to 37 °C (Figure S4). This finding explains why *T. dupontii*, with a largely reduced genome, uses PPIA-derived iron chelators to sequester iron and control iron metabolism in response to temperature reductions.

We observed that PIA-derived iron chelators (1a–6a) in *T. dupontii* were markedly different from those found in common fungi *Aspergillus* species. In *Aspergillus* species, one of the two hydroxamate cores in PIA-derived iron chelators was methylated or acetylated, thereby reducing the efficiency of chelating iron by half (Arai et al. 1981; Yin et al. 2013; Haas 2014). In contrast, both hydroxamate cores in cyclicdipeptide-derived iron chelators from bacteria were available for chelating iron (Cryle et al. 2010; Giessen and Marahiel 2015; Canu et al. 2020). Thus, the iron chelators with two available hydroxamate cores in *T. dupontii* were quite similar to those in bacteria. Furthermore, PPIAs were only found in common mesophilic fungi. The PPIA-derived iron chelators from *T. dupontii* shared the features of both bacteria and common mesophilic fungi.

In summary, our study revealed that *P450S* and *P450L* play an essential role in maintaining a high ratio of Fe^2+^/Fe^3+^ in fungal response to temperature reduction. Our findings suggest that *P450* gene-mediated chemical transformation and their subsequent biological functions regulate iron metabolism, thereby influencing fungal infrastructures and phenotypes during temperature reduction. This provides a mechanistic insight into the biological and ecological functions of the most common *P450* genes in fungal adaption to temperature changes.

## 5. Conclusions

*P450* genes that modify and transform secondary metabolites are known to be hotspots for the generation of diverse and complex metabolites, but their ecological importance remains poorly understood. *Thermomyces dupontii* is a dominant thermophilic fungus with an optimal growth temperature of 45–50 °C and a minimum growth temperature of 37 °C. *T. dupontii* has adapted to environmental temperatures in the biosphere as a key eukaryote decomposer with a sharply reduced genome size, enabling the functions of individual genes to be studied in response to temperature changes. We discovered that two *P450* genes (*P450S* and *P450L*) exist in the cluster for the biosynthesis of PIAs that accumulate in *T. dupontii* in response to temperature changes from 45 °C to 37 °C. Interestingly, *P450L* is a unique fusion gene that encodes two functional domains that were separately encoded by two independent genes in other fungi. These two *P450* genes have evolved to have multifunctions in forming simple PIA-derived iron chelators, bolstering PIAs to complex PPIAs, and yielding effective iron chelators. Moreover, *P450L* has an additional role in the formation of more complex iron chelators derived from new PPIAs. All the P450-upgraded metabolites are involved in maintaining high ratios of Fe^2+^/Fe^3+^, which control iron-mediated mitochondrial contents in mycelia and build up strong and stout conidiophores, thereby facilitating thermophilic fungal conidial survival under cold stress.

## Supplementary Material

Supplemental Material
