# Supplementary material for: Gene fusion and functional diversification of P450 genes facilitate thermophilic fungal adaptation to temperature change
Source: Mycology. 2024 Apr 2;15(3):485–505. doi: 10.1080/21501203.2024.2324993 (PMC11376295; doi:10.1080/21501203.2024.2324993)
Supplement: Supplemental Material [file TMYC_A_2324993_SM8511.docx]

**Figure S1.** Comparison of the content level of a known iron chelator, 11,14-dihydroxylneochinulin E, in two mutants and WT.

**Figure S2.** ECD spectra of compounds **7** (left) and **9** (right).


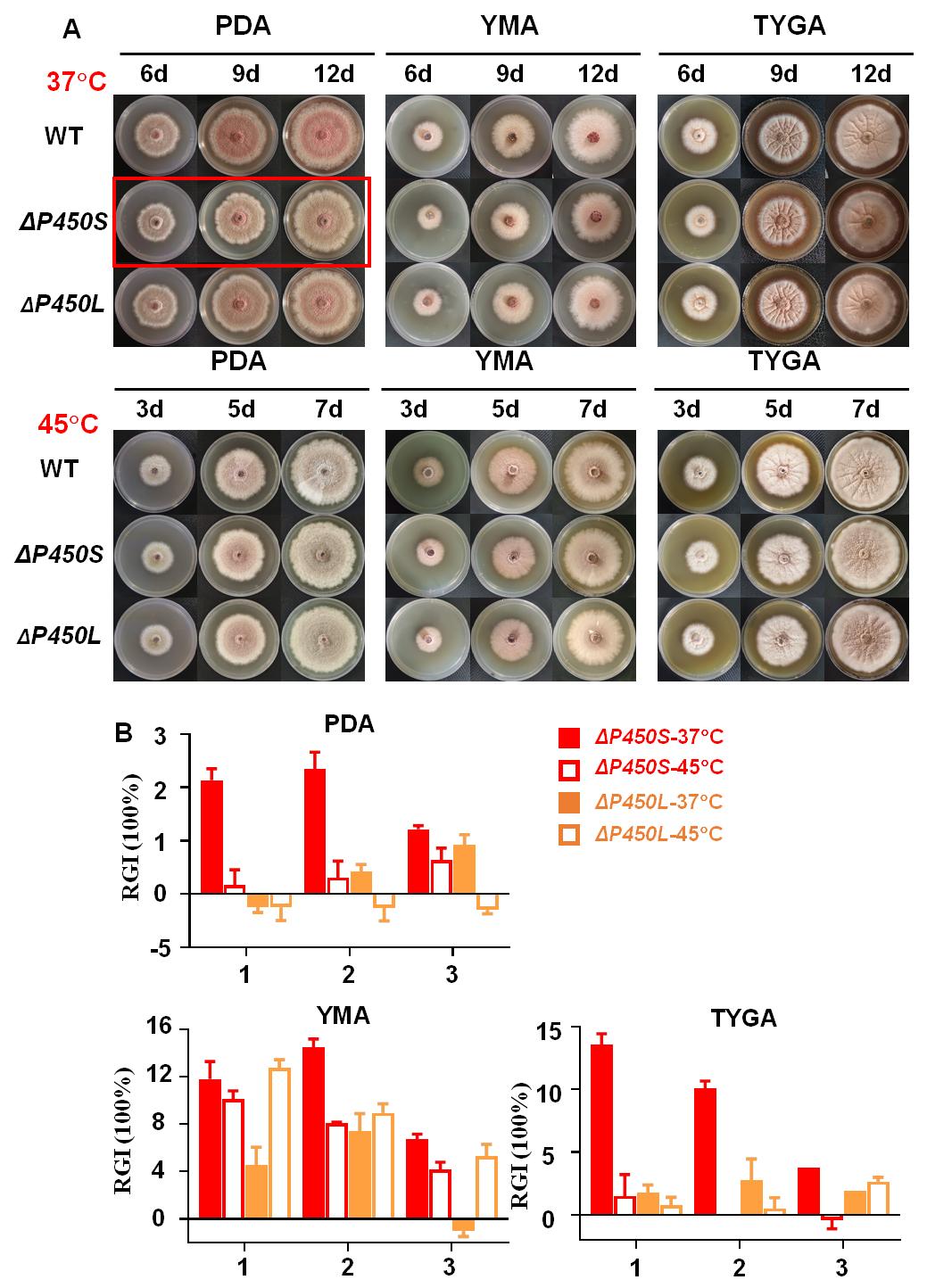


**Figure S3.** The functions of genes *P450S* and *P450L* in fungal growth at relatively low temperatures. (A) Comparison of fungal colony growths between two *Thermomyces dupontii* mutants *ΔP450S* and *ΔP450L*, and WT on PDA, YMA, and TYGA, at 37 °C for 6, 9, and 12 days and at 45 °C for 3, 5, and 7 days. (B) The relative growth inhibition (RGI) of fungal colony growths of mutants *ΔP450S* and *ΔP450L* compared to WT on PDA, YMA, and TYGA, at 37 °C for 6, 9, and 12 days and at 45 °C for 3, 5, and 7 days. Two mutants Δ*P450S* and Δ*P450L* displayed retarded growth at 37 °C.


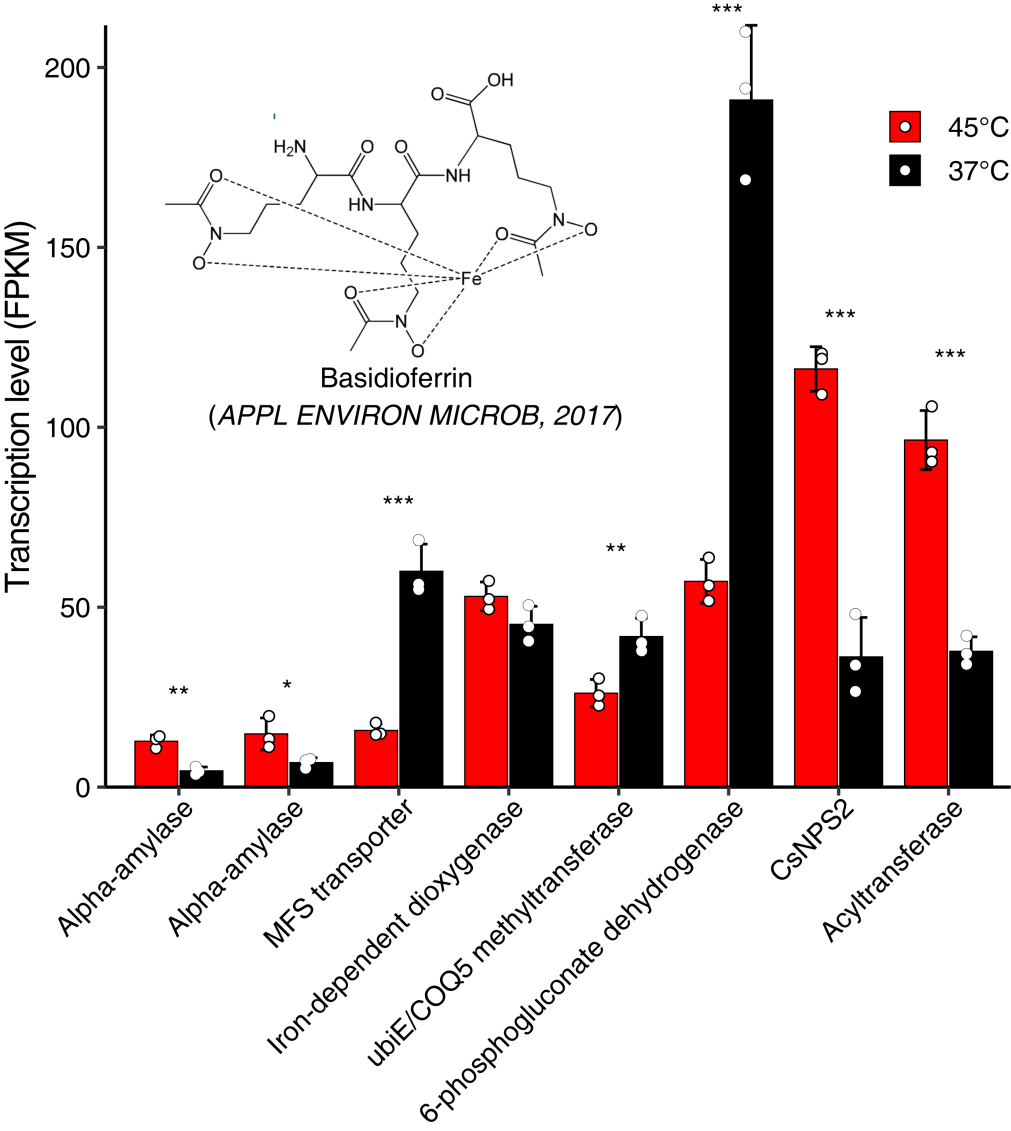


**Figure S4.** Transcriptional levels of genes in the cluster for basidioferrin biosynthesis in WT at 37 °C and 45 °C.


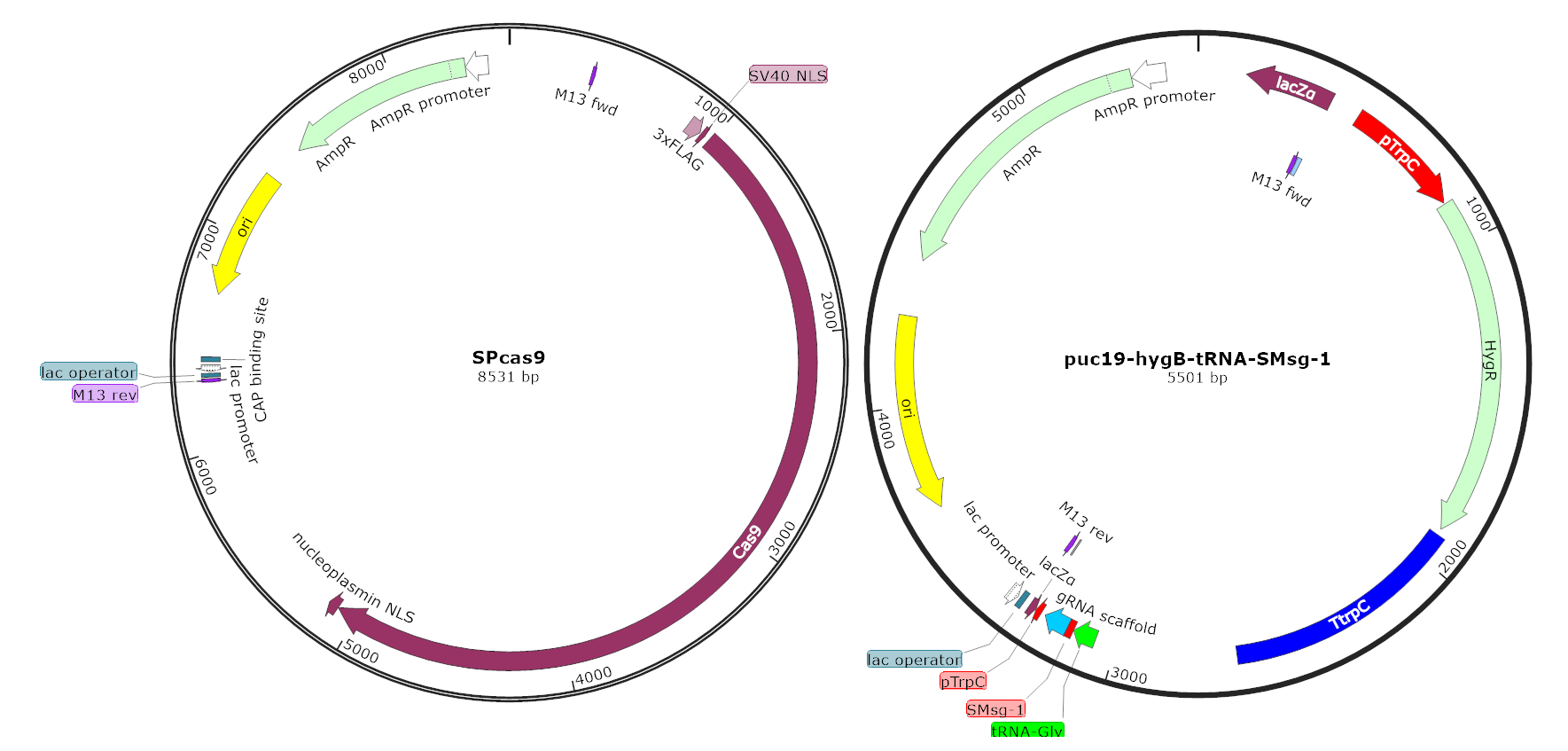


**Figure S5**. Plasmid map used in the study.


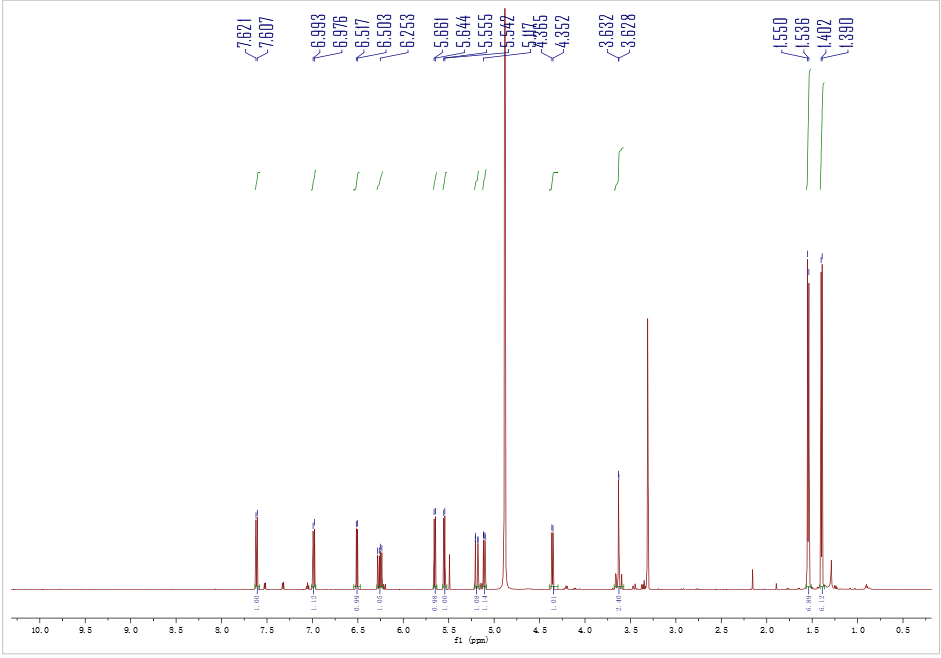


**Figure S6.** ^1^H NMR spectrum of metabolite **7**.


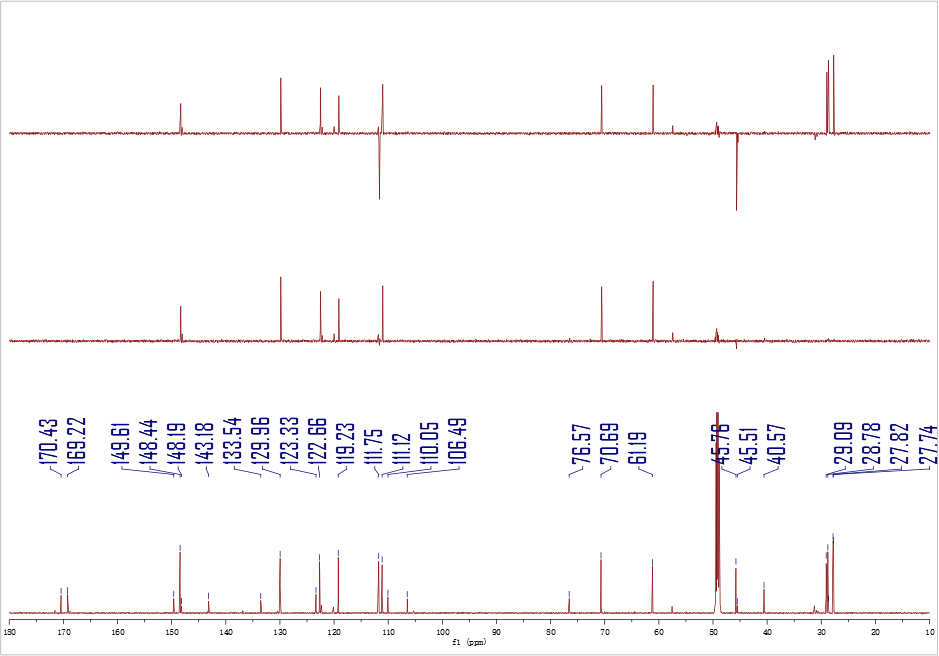


**Figure S7.** ^13^C NMR spectrum of metabolite **7**.


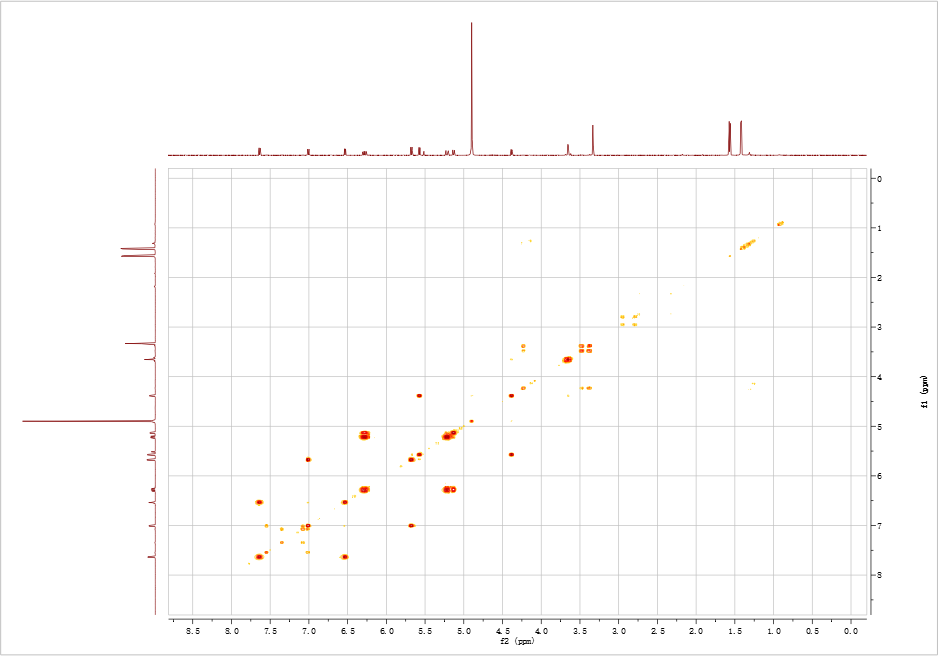


**Figure S8.** COSY spectrum of metabolite **7**.


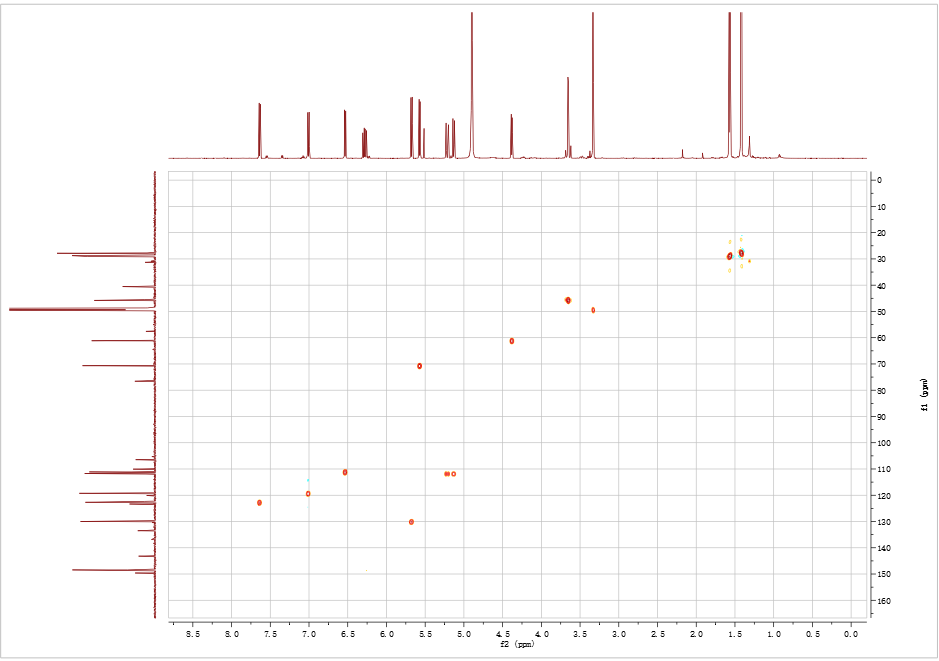


**Figure S9.** HSQC spectrum of metabolite **7**.


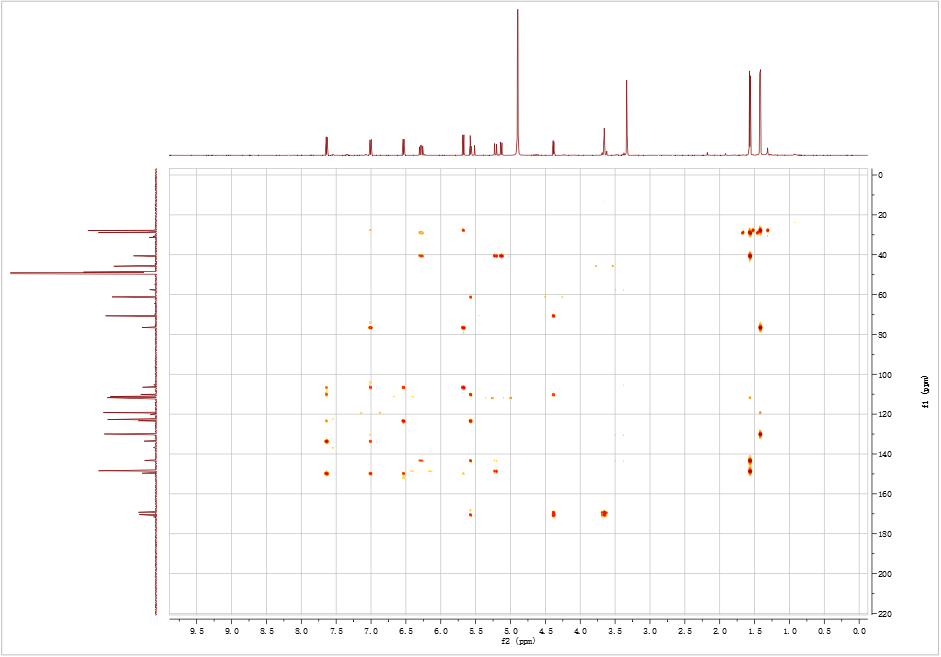


**Figure S10.** HMBC spectrum of metabolite **7**.


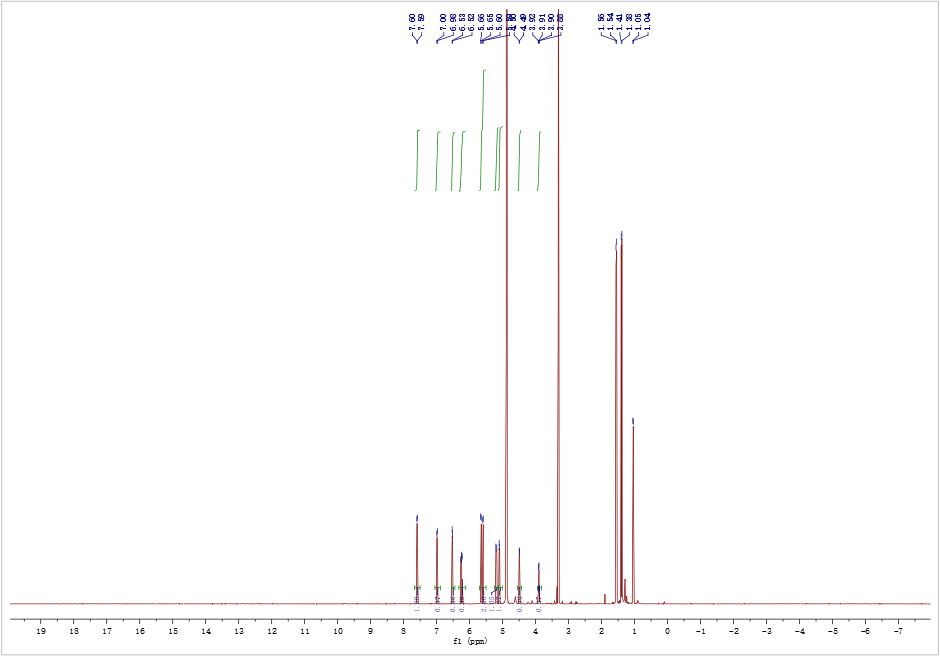


**Figure S11.** ^1^H NMR spectrum of metabolite **8**.


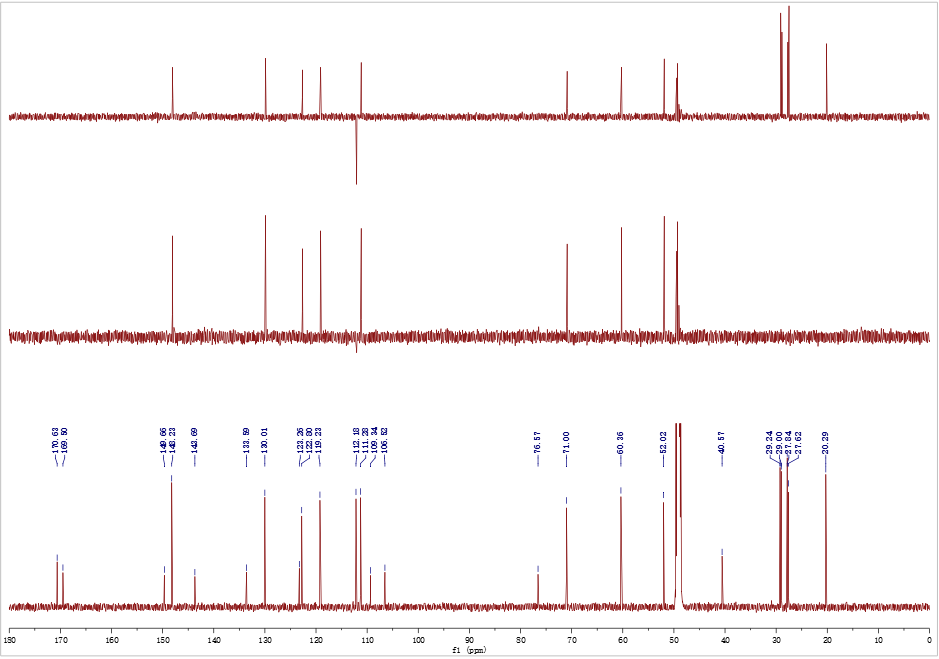


**Figure S12.** ^13^C NMR spectrum of metabolite **8**.


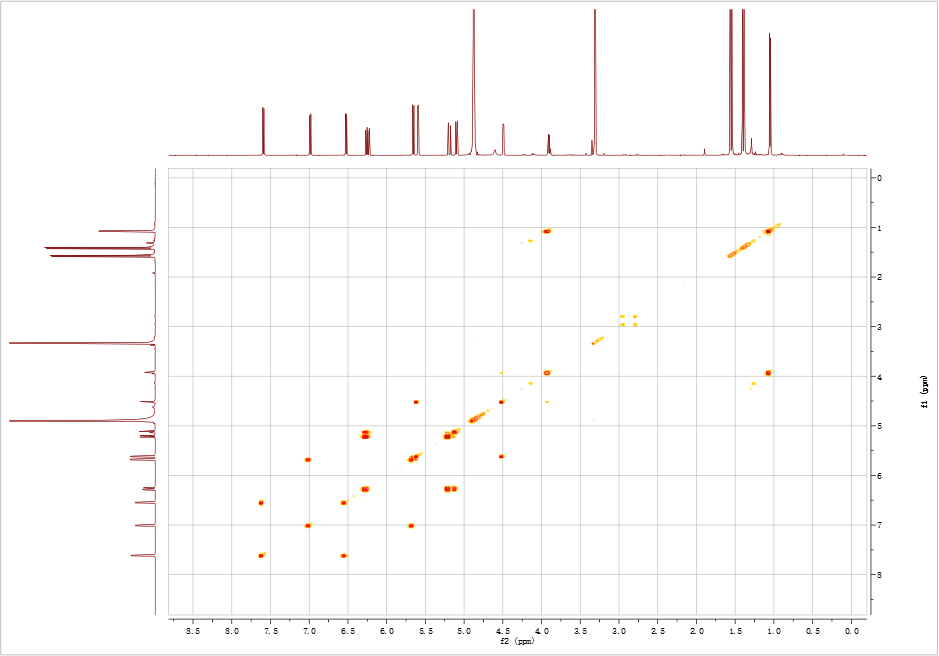
**Figure S13.** COSY spectrum of metabolite **8**.


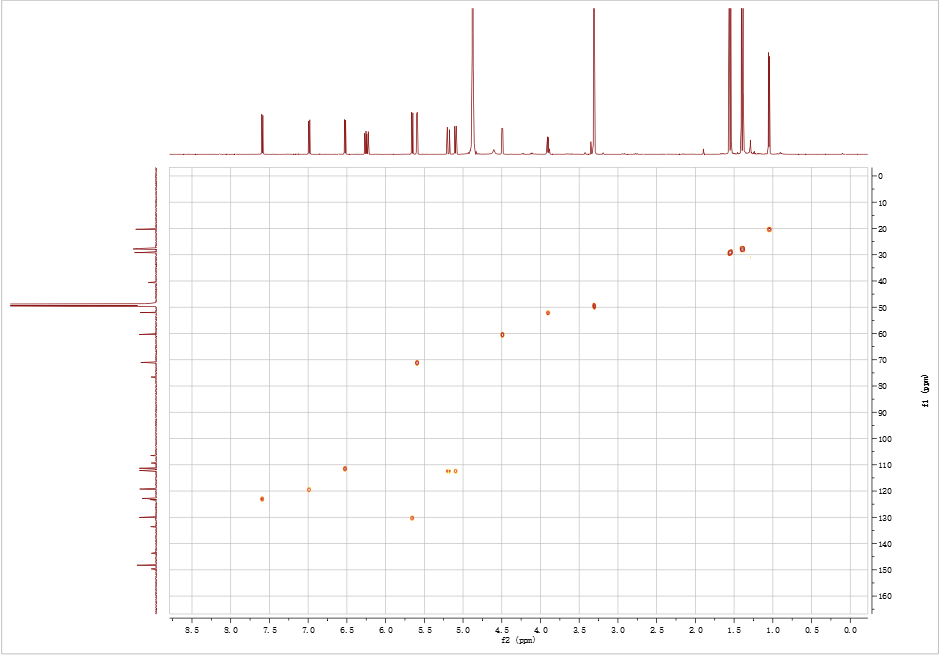


**Figure S14.** HSQC spectrum of metabolite **8**.


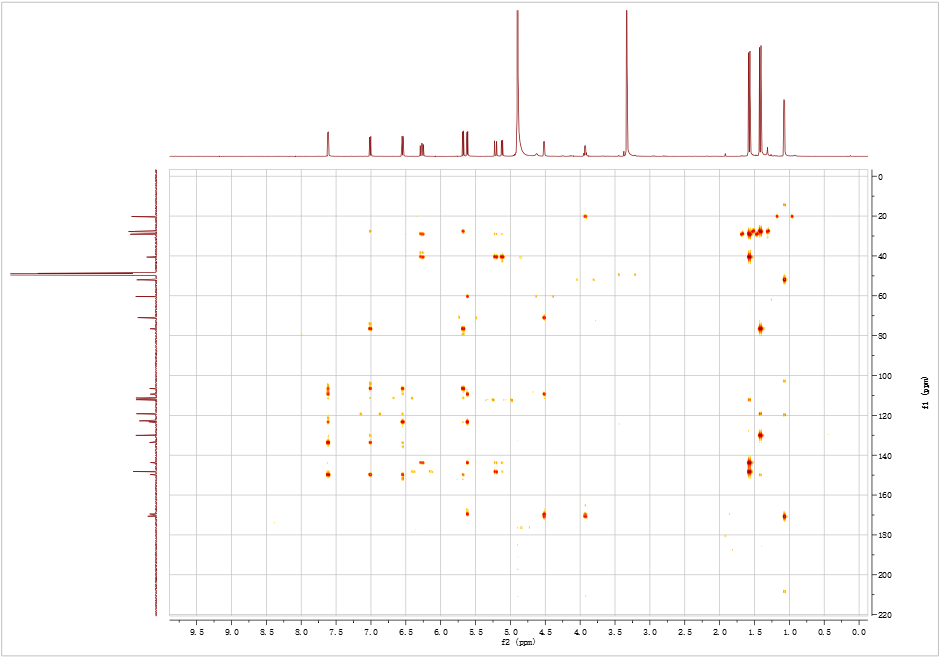


**Figure S15.** HMBC spectrum of metabolite **8**.


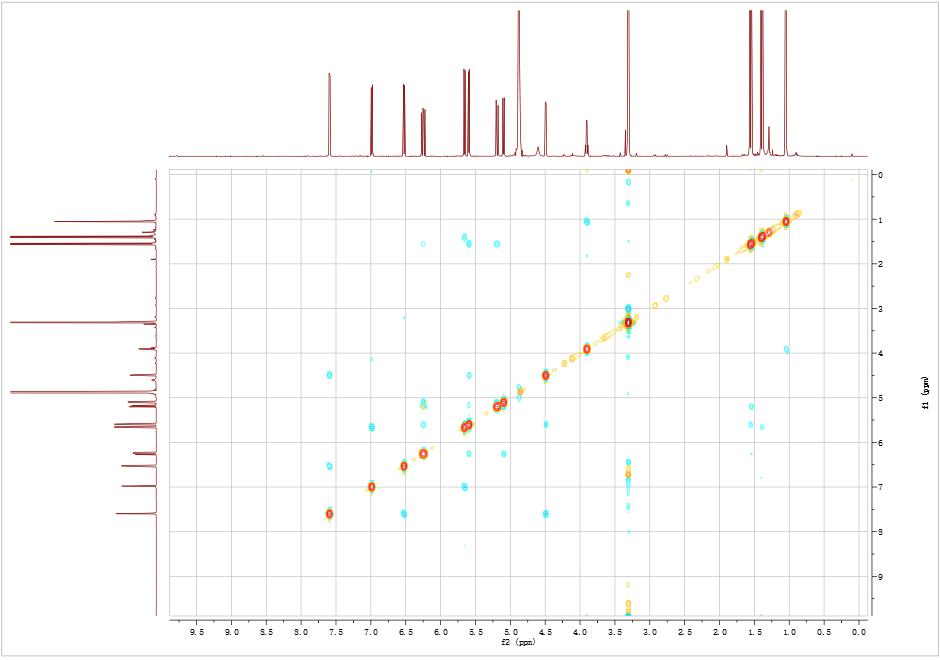


**Figure S16.** NOESY spectrum of metabolite **8**.


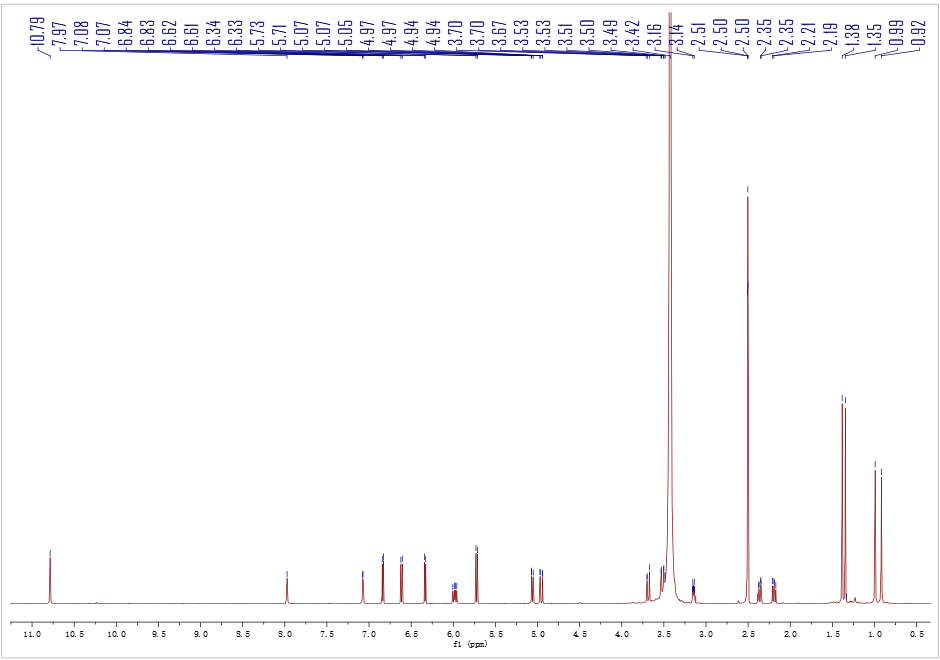


**Figure S17.** ^1^H NMR spectrum of metabolite **9**.


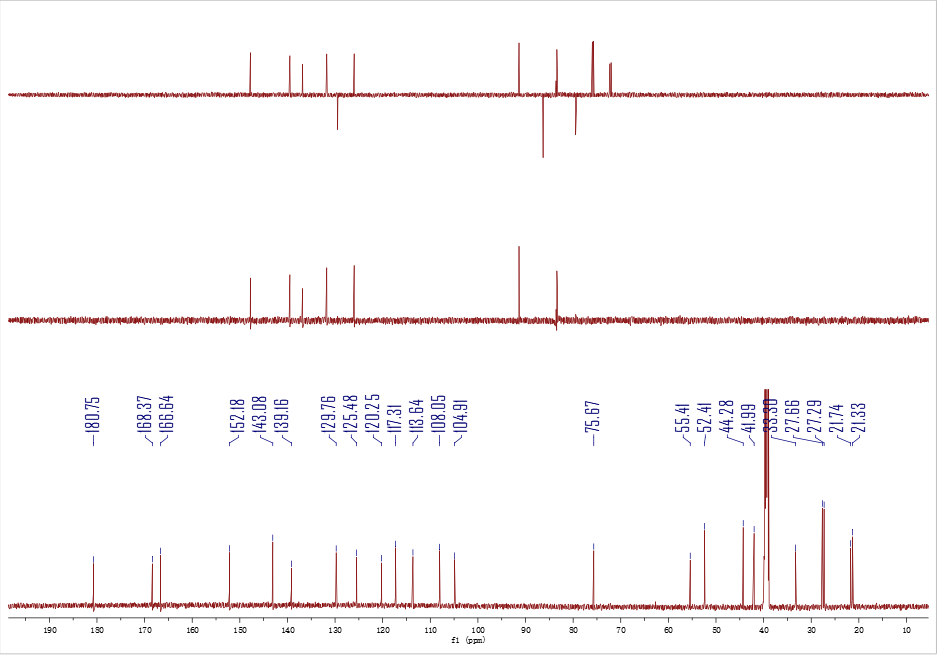


**Figure S18.** ^13^C NMR spectrum of metabolite **9**.


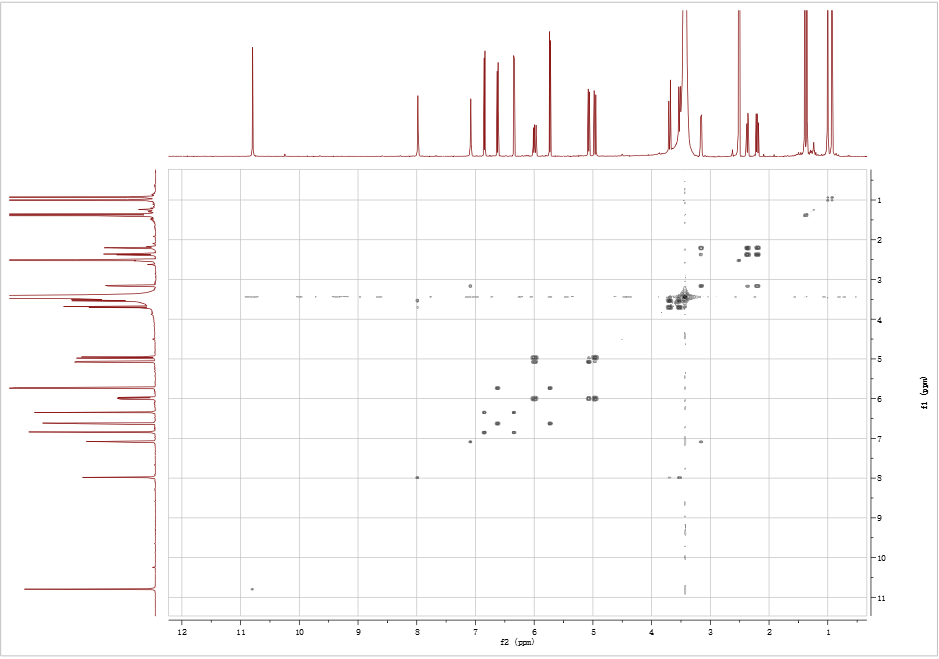


**Figure S19.** COSY spectrum of metabolite **9**.


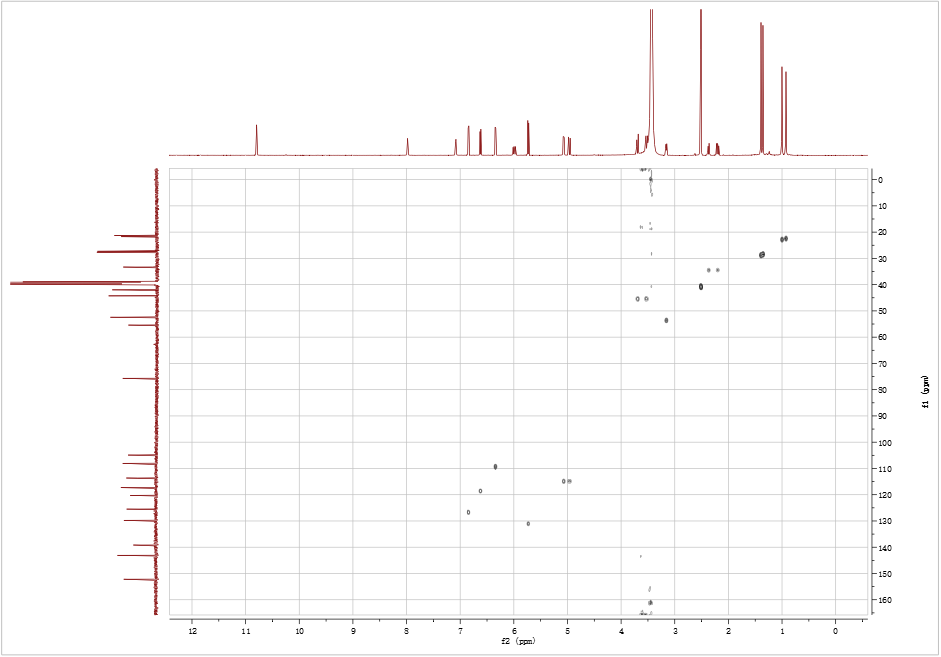


**Figure S20.** HSQC spectrum of metabolite **9**.


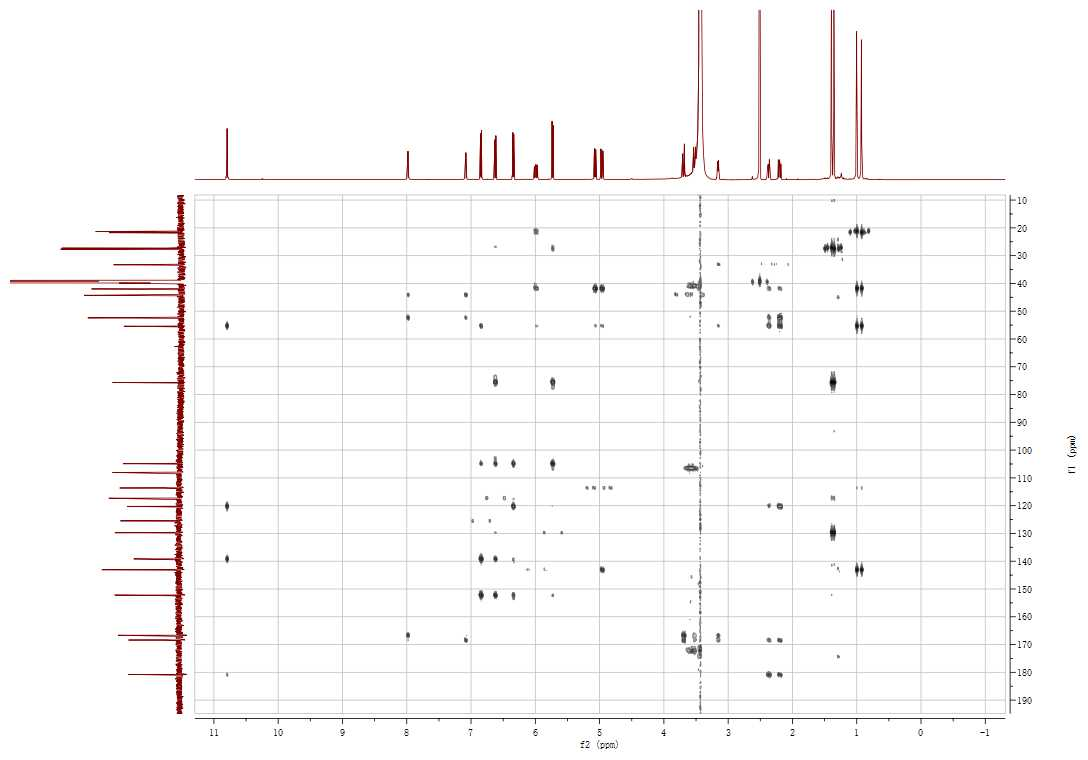


**Figure S21.** HMBC spectrum of metabolite **9**.


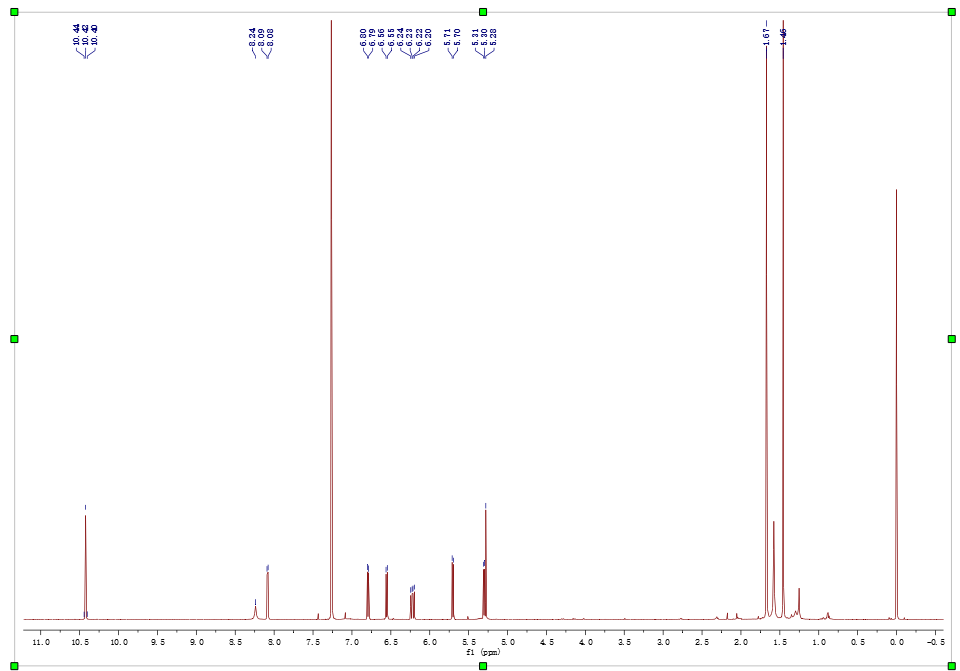


**Figure S22.** ^1^H NMR spectrum of metabolite **11**.


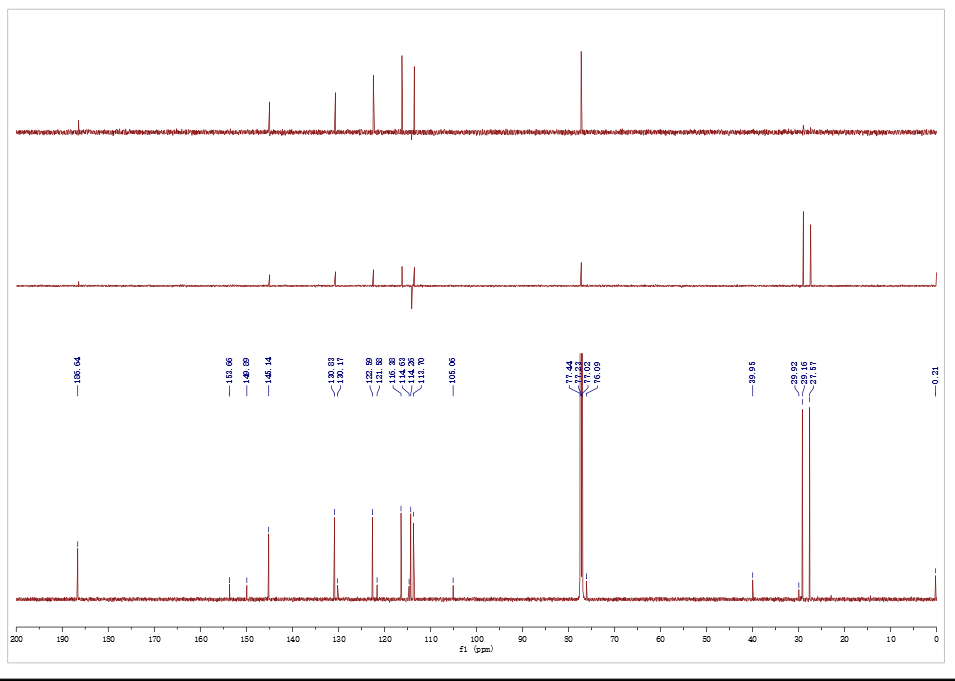


**Figure S23.** ^13^C NMR spectrum of metabolite **11**.


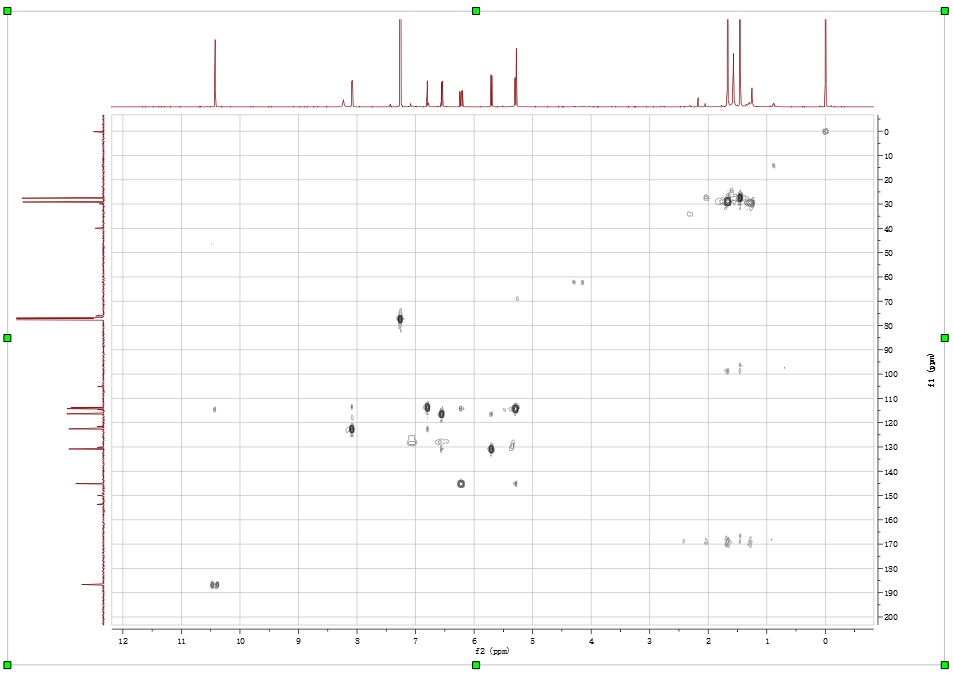


**Figure S24.** HSQC spectrum of metabolite **11**.


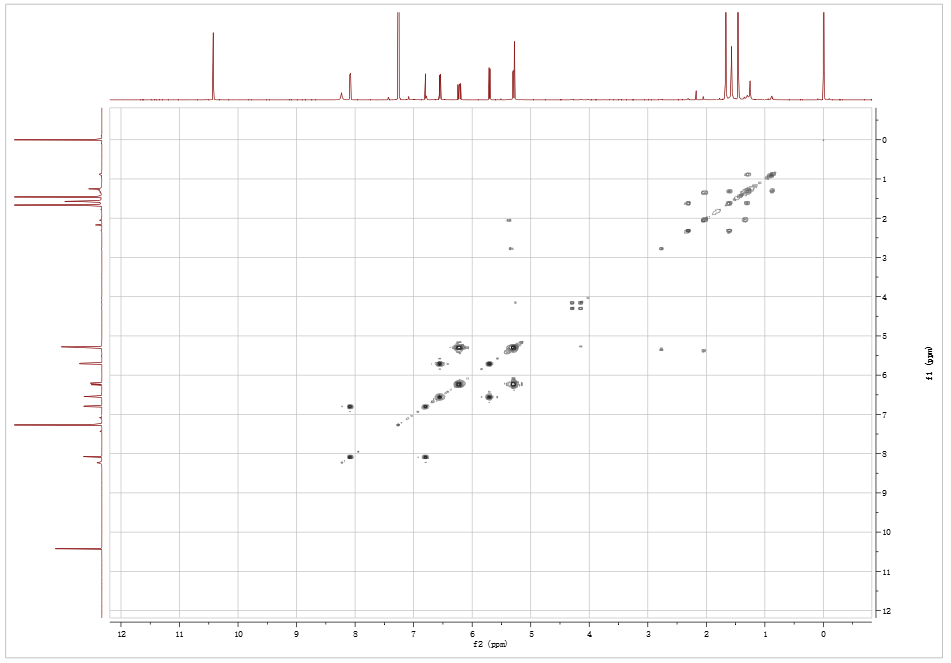


**Figure S25.** ^1^H-^1^H COSY spectrum of metabolite **11**.


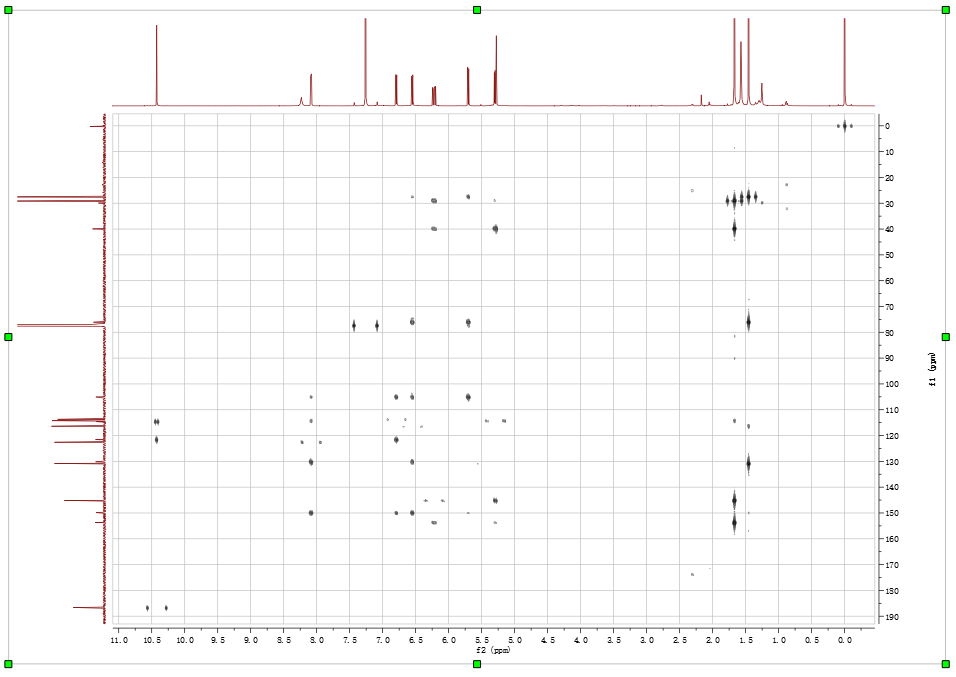


**Figure S26.** HMBC spectrum of metabolite **11**.

Talathermophilin **G** (**7**): Colorless oil; C_23_H_27_N_3_O_4_; [α]^20.7°^ + 22.14 ° (c 0.14, MeOH); IR (KBr) ν_max_ 3433.3, 3385.7, 3268.1, 3086.5, 3053.1, 2973.6, 2924.3, 2855.1, 1675.1, 1583.9, 1560.4, 1493.4, 1444.9, 1380.4, 1358.5, 1322.5, 1308.6, 1248.5, 1217.5, 1193.8, 1166.7, 1120.6, 1065.4, 1046.0, 996.9, 932.3, 922.6, 847.0, 812.6, 780.5, 761.3, 736.9, 718.6, 687.7, 660.6, 631.7, 617.1, 586.8, 536.7, 514.4, 492.5, 439.4, 426.2 cm ^-1^; ^1^H NMR and ^13^C NMR see Tables 1 and 2; Negative HRESI-MS *m/z* 408.1932 [M-H]^-^, calculated 408.1929 for C_23_H_26_N_3_O_4_.

Talathermophilin **H** (**8**): Colorless oil; C_24_H_29_N_3_O_4_; ^1^H NMR and ^13^C NMR see Tables 1 and 2; Positive HRESI-MS *m/z* [M+H]^+^, calculated 424.2230 for C_24_H_30_N_3_O_4_.

Talathermophilin **I** (**9**): Colorless oil, C_23_H_27_N_3_O_4_; IR (KBr) ν_max_ 3421.7, 2975.5, 2928.2, 1705.2, 1679.4, 1644.9, 1456.5, 1383.5, 1309.1, 1216.9, 1156.5, 1119.7, 1084.1, 1047.5, 923.8, 878.7, 732.8, 669.0, 565.5, 484.9 cm ^-1^; ^1^H NMR and ^13^C NMR see Tables 1 and 2; Positive HRESI-MS *m/z* 432.1895 [M+Na]^+^, calculated 432.1894 for C_23_H_27_N_3_O_4_Na.

Compound **11**: C_24_H_29_N_3_O_4_. Positive HRESI-MS *m/z* [M+H]^+^ 424.2236, calculated 424.2232 for C_24_H_30_N_3_O_4_. ^1^H-NMR (600 MHz, CDCl_3_) δ_H_: 8.24 (1H, brs, NH-1), 8.08 (1H, d, J = 8.3 Hz, H-4), 6.79 (1H, d, J = 8.3 Hz, H-5), 6.22 (1H, dd, J = 17.6, 10.6 Hz, H-11), 5.31 (1H, d, J = 17.6 Hz, H-12a), 5.28 (1H, d, J = 10.6 Hz, H-12b), 1.67 (6H, s, H-13,14), 6.55 (1H, d, J = 9.7 Hz, H-15), 5.70 (1H, d, J = 9.7 Hz, H-16), 1.46 (6H, s, H-18,19), 10.42 (1H, s, H-20); ^13^C-NMR (150 MHz, CDCl_3_) δ_C_: 153.7 (C-2), 121.6 (C-3), 122.6 (C-4), 113.7 (C-5), 149.9 (C-6), 105.1 (C-7), 130.2 (C-8), 114.6 (C-9), 40.0 (C-10), 145.1 (C-11), 114.3 (C-12), 29.2 (C-13,14), 116.4 (C-15), 130.8 (C-16), 76.1 (C-17), 27.6 (C-18,19), 186.6 (C-20).

**Table S1.** The ^1^ H (400 MHz, CD_3_OD) data for **7**−**9**.

| No | **7** | **8** | **9** |
| --- | --- | --- | --- |
| 1 |  |  | 10.79 s |
| 4 | 7.61 d 8.4 | 7.60 d, 8.4 | 6.84 d 7.6 |
| 5 | 6.51 d 8.4 | 6.53 d, 8.4 | 6.34 d, 7.6 |
| 11 | 6.25 dd 10.4, 17.8 | 6.25 dd 10.4, 17.8 | 5.99 dd 11.5, 17.9 |
| 12 | 5.21 d 17.8  5.11 d 10.6 | 5.19 d 17.8  5.10 d 10.6 | 5.06 d 11.5  4.96 d 17.9 |
| 13 | 1.55 s | 1.56 s | 0.99 s |
| 14 | 1.53 s | 1.54 s | 0.92 s |
| 15 | 5.55 d 7.4 | 5.60 d 7.2 | 2.36 dd 14.6, 3.6  2.19 dd 10.2, 14.6 |
| 16 | 4.36 d 7.4 | 4.50 d 7.0 | 3.15 dt 9.3, 2.8 |
| 18 |  |  | 7.97 s |
| 19 | 3.62 d 2.8 | 3.92 q 6.9 | 3.68 d 18.0  3.52 dd 18.2, 2.8 |
| 21 |  |  | 7.08 s |
| 22 | 6.98 d 9.9 | 6.99 d 9.9 | 6.62 d 10.4 |
| 23 | 5.65 d 9.9 | 5.66 d 9.9 | 5.72 d 10.4 |
| 25 | 1.40 s | 1.41 s | 1.38 s |
| 26 | 1.39 s | 1.38 s | 1.36 s |
| 27 |  | 1.05 d, 6.9 |  |

**Table S2.** The ^13^C NMR (100 MHz, CD_3_OD) data for **7**−**9**.

| No | **7** | **8** | **9** |
| --- | --- | --- | --- |
| 2 | 143.18, C | 143.69, C | 180.75, C |
| 3 | 110.05, C | 109.34, C | 55.41, C |
| 4 | 122.66, CH | 122.80, CH | 125.48, CH |
| 5 | 111.12, CH | 111.28, CH | 108.05, CH |
| 6 | 149.61, C | 149.66, C | 152.18, C |
| 7 | 106.49, C | 106.52, C | 104.91, C |
| 8 | 133.54, C | 133.59, C | 139.16, C |
| 9 | 123.33, C | 123.26, C | 120.25, C |
| 10 | 40.57, C | 40.57, C | 41.99, C |
| 11 | 148.44, CH | 148.26, CH | 143.08, CH |
| 12 | 111.75, CH_2_ | 112.18, CH_2_ | 113.64, CH_2_ |
| 13 | 29.09, CH_3_ | 29.24, CH_3_ | 21.74, CH_3_ |
| 14 | 28.78, CH_3_ | 29.00, CH_3_ | 21.33, CH_3_ |
| 15 | 70.69, CH | 71.00, CH | 33.30, CH_2_ |
| 16 | 61.19, CH | 60.36, CH | 52.41, CH |
| 17 | 170.43, C | 170.63, C | 168.37, C |
| 19 | 45.78, CH_2_ | 52.05, CH | 44.28, CH_2_ |
| 20 | 169.22, C | 169.50, C | 166.64, C |
| 22 | 119.23, CH | 119.23, CH | 117.31, CH |
| 23 | 129.96, CH | 130.01, CH | 129.76, CH |
| 24 | 76.57, C | 76.57, C | 75.67, C |
| 25 | 27.82, CH_3_ | 27.84, CH_3_ | 27.66, CH_3_ |
| 26 | 27.74, CH_3_ | 27.62, CH_3_ | 27.29, CH_3_ |
| 27 |  | 20.29, CH_3_ |  |

**Table S3.** Primers used in this study.

| **Primer name** | **Sequence (5' to 3')** | **Pam** | **Usage** |
| --- | --- | --- | --- |
| P450Lsg-F | ATACGGAGAGCGCCTCGACCGTTTTAGAGCTAGAAATAGCAAGTTAAAATAAGG | CGG | Amplification of sgRNA, The N20 sequence of the gene of interest is marked in red |
| P450Lsg-R | GGTCGAGGCGCTCTCCGTATLTGCATGATCCGCGAATCG |  |  |
| P450Ssg-F | TCACTCGCATCGAACTAGAGGTTTTAGAGCTAGAAATAGCAAGTTAAAATAAGG | CGG |  |
| P450Ssg-R | CTCTAGTTCGATGCGAGTGATGCATGATCCGCGAATCG |  |  |
| ZLyz-F | TCTCCTTGCATGCACCATTC | —— | *p-tRNAGly-sgRNA* plasmid N20 sequence validation |
| ZLyz-R | CCCCAGGCTTTACACTTTATGC |  |  |
| P450Lyz-F | GAAGCTCGAGGGCCGTTTG | —— | Positive transformants of the target gene verify primers |
| P450Lyz-R | GCCTTACGGACCACGATGG | —— |  |
| P450Syz-F | AGTCTCCTTGTATACCGAGG | —— |  |
| P450Syz-R | TTTCATTTGCATGGGCAGC | —— |  |
